# Recent Insights into the Contribution of the Changing Hypertrophic Chondrocyte Phenotype in the Development and Progression of Osteoarthritis

**DOI:** 10.3389/fbioe.2018.00018

**Published:** 2018-03-19

**Authors:** Ellen G. J. Ripmeester, Ufuk Tan Timur, Marjolein M. J. Caron, Tim J. M. Welting

**Affiliations:** ^1^Laboratory for Experimental Orthopedics, Department of Orthopedic Surgery, Maastricht University Medical Center, Maastricht, Netherlands

**Keywords:** chondrocytes, osteoarthritis, hypertrophy, cartilage, phenotype, endochondral ossification

## Abstract

Osteoarthritis (OA) is an extremely prevalent age-related condition. The economic and societal burden due to the cost of symptomatic treatment, inability to work, joint replacement, and rehabilitation is huge and increasing. Currently, there are no effective medical therapies that delay or reverse the pathological manifestations of OA. Current treatment options are, without exception, focused on slowing down progression of the disease to postpone total joint replacement surgery for as long as possible and keeping the associated pain and joint immobility manageable. Alterations in the articular cartilage chondrocyte phenotype might be fundamental in the pathological mechanisms of OA development. In many ways, the changing chondrocyte phenotype in osteoarthritic cartilage resembles the process of endochondral ossification as seen, for instance, in developing growth plates. However, the relative contribution of endochondral ossification to the changing chondrocyte phenotype in the development and progression of OA remains poorly described. In this review, we will discuss the current knowledge regarding the cartilage endochondral phenotypic changes occurring during OA development and progression, as well as the molecular and environmental effectors driving these changes. Understanding how these molecular mechanisms determine the chondrocyte cell fate in OA will be essential in enabling cartilage regenerative approaches in future treatments of OA.

## Introduction

Osteoarthritis (OA) is the most common degenerative joint disorder worldwide and its incidence rises with age (Loeser et al., 2012). The economic and societal burden due to costs of symptomatic treatment, inability to work, joint replacement surgery (and coinciding implant infections), rehabilitation, and social isolation is huge (Bijlsma et al., 2011; Le et al., 2012). Identifying the main molecular mechanisms by which OA is initiated and progresses is still one of the biggest challenges in this field. However, our current knowledge teaches us that the initial trigger for developing OA is multifactorial with risk factors, including obesity, diabetes, genetics, and trauma (Felson et al., 2000). Despite the diversity of initial triggers OA disease progression follows a predictable cell biological progression. While pain experience, joint immobility, and speed of disease progression are some of the few patient-variable parameters. Depending on the OA-stage, interventions are mainly based on alleviating chronic pain and preserving joint mobility by visco-supplementation, and physiotherapy to postpone joint replacement surgery (Lo et al., 2003; Zhang et al., 2010; Page et al., 2011). While there is a lack of treatment options that are disease-modifying and improve joint-tissue homeostasis. Substantial effort is required to identify targetable pathways or individual factors that alter the diseased cartilage phenotype in OA.

A prominent feature of OA is cartilage degradation. Other joint structures, such as the synovium, Hoffa’s fat pad (HFP), meniscus, and subchondral bone have been demonstrated to experience OA-specific pathologic changes. Changes in these joint structures include not only infiltration of active immune cells in the synovium and HFP, but also fibrillation of the meniscus and sclerosis of the subchondral bone. Together, these changes lead to a loss of joint mobility and function, accompanied by chronic pain (Loeser et al., 2012).

From a biochemical perspective, the cartilage degradation observed in OA has been attributed to an elevated production of proteolytic enzymes, such as matrix metalloprotease 13 (MMP13) and aggrecanases, such as a disintegrin and metalloproteinase with thrombospondin motifs (ADAMTS) 4 and 5. These degrade important cartilage matrix components, such as type II collagen (COL2A1) and aggrecan (Hunter, 2011). In addition to elevated production of proteolytic enzymes in OA cartilage, other observed features in OA cartilage include the expression of chondrocyte hypertrophic markers [such as type 10 collagen (COL10A1)] (Little et al., 2009), vascularization, and focal calcification. Since, these features resemble the endochondral ossification process that occurs in the hypertrophic zone of the growth plate, it has been hypothesized that OA is a disease characterized by ectopic recapitulation of the endochondral ossification process (Dreier, 2010; Pitsillides and Beier, 2011; van der Kraan and van den Berg, 2012).

Post-developmental healthy articular cartilage homeostasis is thought to be “protected” against hypertrophic or catabolic changes by several pathways employing soluble mediators, including BMP-, TGF-β-, and hedgehog signaling (Dreier, 2010; Pitsillides and Beier, 2011). These pathways transcriptionally control the chondrocyte phenotype by tuning the activity and levels of major chondrocyte phenotype-determining downstream transcription factors, such as SOX9, RUNX2, and SMADs (van der Kraan and van den Berg, 2012). Many risk factors for developing OA are thought to (in)directly influence the activity of these pathways, and thus ultimately resulting in a changing chondrocyte phenotype that becomes disposed to entering endochondral ossification. This may place, besides the local inflammatory condition (among others caused by synovitis), the chondrocyte/cartilage differentiation status central to the progression, or cause of OA.

The relation between the chondrocyte/cartilage differentiation status and OA development and -progression has been well-described and was acknowledged in the past by a number of excellent reviews (Dreier, 2010; Pitsillides and Beier, 2011; van der Kraan and van den Berg, 2012). In this review, we present an overview of the literature from the past 10 years, describing recent insights in the contribution of endochondral ossification-related processes in OA disease development and -progression. We conducted a PubMed literature search including papers from the past 10 years discussing endochondral ossification and its accompanying processes also occurring in OA disease progression. Molecular insight in the role of chondrocyte hypertrophic processes involved in OA initiation and progression is expected to provide valuable information for drug development targeting these processes for OA disease modification.

## Review Procedure

**Figure 1:**
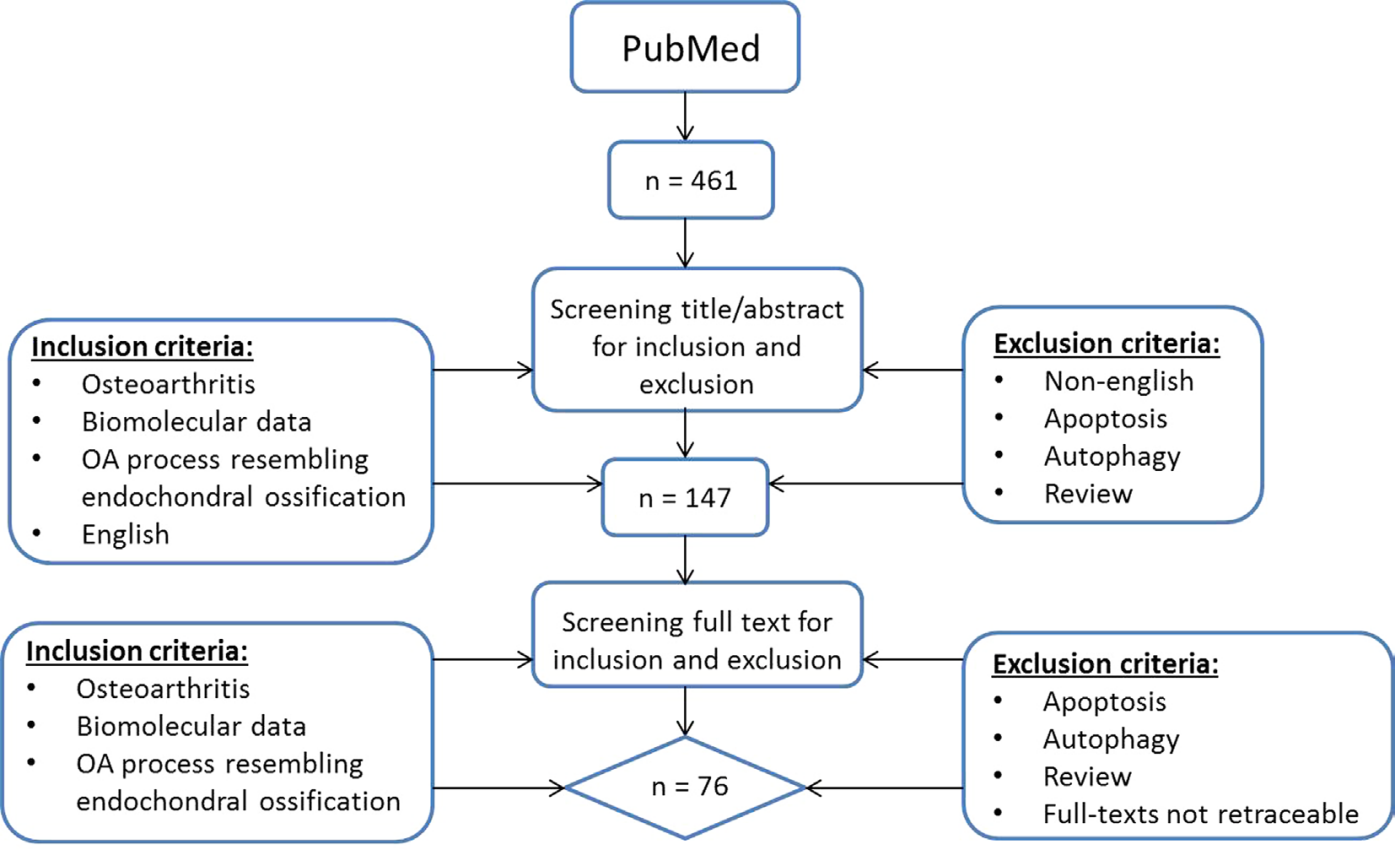
Literature search flowchart.

To provide a current status on the role of hypertrophic changes in OA we searched for English manuscripts from the past 10 years on PubMed using the following search strategy (Figure 1): (OA OR osteoarthrosis OR osteoarthritis OR non-inflammatory arthritis OR degenerative arthritis OR osteoarthritic) AND (hypertrophic OR hypertrophy OR terminal differentiation OR hypertrophic differentiation OR end stage differentiation, OR endochondral ossification OR chondrocyte hypertrophy OR transdifferentiation OR mineralization OR mineralisation OR mineralized OR mineralised OR calcification) and chondrocyte. At May 3rd, 2017 a total of 461 papers were found and screened *via* title and abstract by two separate observers (ER and UT) using the following inclusion criteria: papers describing osteoarthritis, biomolecular data, and literature describing an OA process that resembles endochondral ossification in the title and abstract. Articles referring to apoptosis and autophagy were excluded as we wanted to focus on processes occurring during OA initiation and progression and we considered apoptosis and autophagy as end-stage processes. Besides apoptosis and autophagy, reviews were also excluded. When there was a discrepancy in paper selection, consensus was reached with all authors. This resulted in a short-list of 147 articles whose full-text was manually screened by four observers (ER, UT, MC, and TW) using the same inclusion criteria. Papers with missing full-texts were excluded. This resulted in a total of 76 papers being included in this review (Figure 1).

## Results

Our search yielded a diverse range of publications from the past 10 years confirming earlier reports describing endochondral cellular phenotypic changes in OA cartilage and describing associations of these endochondral cellular phenotypic changes with the development and progression of OA. During our search, we noticed that the majority of the papers could be classified into signaling pathways known to be involved in endochondral ossification, such as *Wnt-, Ihh/PTHrP-, TGF-*β*-, MAP-kinases, FGF-, Notch signaling, inflammatory signaling, and hypoxia-associated signaling pathways*. Besides these pathways also papers describing processes, such as angiogenesis and matrix mineralization were found. These pathways and processes involved in endochondral ossification will be described now separately and new insights from the selected literature will be discussed with respect to these pathways.

### Wnt Signaling

**Figure 2:**
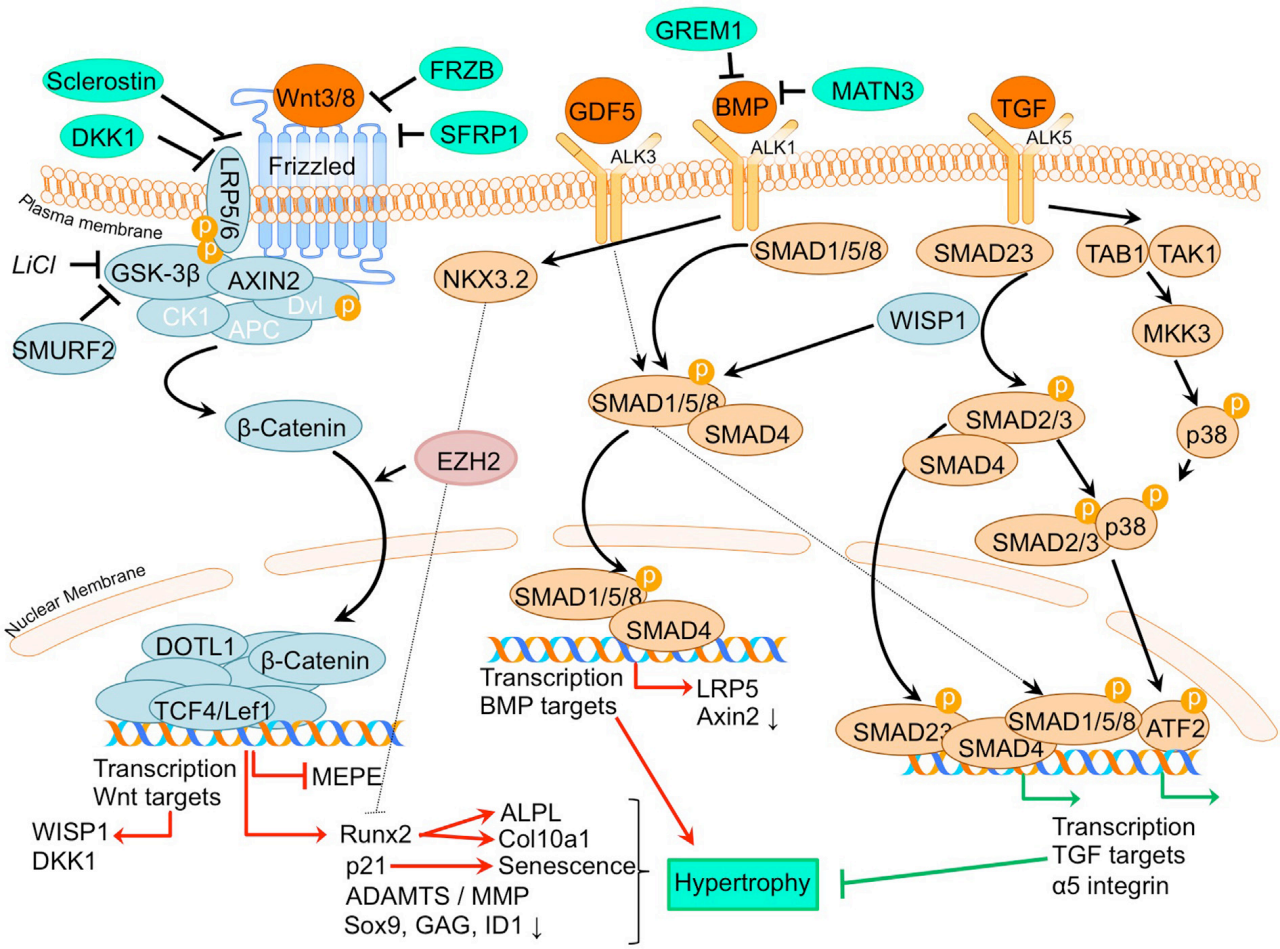
Overview of the canonical Wnt- and TGFβ superfamily signaling pathways and newly acquired insights into their relation to the development of the osteoarthritic (OA) chondrocyte hypertrophic phenotype. The canonical pathway was updated with recent insights in the role of Wnt- and TGFβ superfamily signaling in the hypertrophic switch occuring during OA development. In green pro-chondrogenic relations are shown, while pro-hypertrophic relations are depicted in red.

A thoroughly studied pathway involved in chondrocyte differentiation and hypertrophy is Wnt signaling. The canonical Wnt/β-catenin pathway (Wu et al., 2009; Borzi et al., 2010; Pitsillides and Beier, 2011; Castano Betancourt et al., 2012; Facchini et al., 2012; Papathanasiou et al., 2012; Leijten et al., 2013; van den Bosch et al., 2014; Guidotti et al., 2015; Chen et al., 2016; Staines et al., 2016) is activated by binding one of the canonical Wnt ligands to a Frizzled family receptor and an LRP5/6 co-receptor, which passes the signal *via* GSK-3β to inhibit the β-catenin destruction complex, causing an accumulation of β-catenin in the cytoplasm and eventual its translocation into the nucleus (MacDonald et al., 2009). This enables transcriptional coactivation with TCF/LEF transcription factors which are active in the transcription of Wnt-target genes, such as RUNX2 (MacDonald et al., 2009). In our review procedure, we did not find literature regarding non-canonical Wnt signaling that matched the search criteria. An overview of newly acquired insights into this pathway and its involvement in development of OA chondrocyte hypertrophy is provided in Table 1a and Figure 2.

**Table 1:** (A–K) Hypertrophy associated-factors.

Abbreviation	Name	Reference
**A: Wnt/β-catenin**		
AXIN2	Axis inhibition protein 2	Chen et al. (2016)
S-oxo-dG	8-Oxo-2’-deoxyguanosine	Guidotti et al. (2015)
DKK1	Dickkopf 1 homolog	Leijten et al. (2013)
DMP1	Dentin matrix acidic phosphoprotein 1	Staines et al. (2016)
DOT1L	Disruptor of teiomeric silencing 1-like	Castano Betancourt et al. (2012)
EZH2	Enhancer of zeste homolog 2	Chen et al. (2016)
FR1ZB	Frizzled-related protein	Leijten et al. (2013)
GADD45b	Growth arrest and DNA damage inducible beta	Ijiri et al. (2008) and Guidotti et al. (2015)
GSK-3β	Glycogen synthase kinase 3 beta	Guidotti et al. (2015)
LEF1	Lymphoid enhancer-binding factor 1	Papathanasiou et al. (2012) and Chen et al. (2016)
LRP5	Low-density lipoprotein receptor-related protein 5	Papathanasiou et al. (2012)
LRP6	Low-density lipoprotein receptor-related protein 6	Papathanasiou et al. (2012)
MEPE1	Matrix extracellular phosphoglycoprotein p21	Staines et al. (2016)
PAS	Periodic acid-Schiff staining	Guidotti et al. (2015)
PHEX	Phosphate-regulating neutral endopeptidase, X-linked	Staines et al. (2016)
SAβ Galactosidase	Senescence-associated beta-gal actosidase	Guidotti et al. (2015)
SFRP1	Secreted frizzled-related protein 1	Chen et al. (2016)
SMURF2	SMAD-specific E3 ubiquitin protein ligase 2	Wu et al. (2009)
SOST	Sclerostin	Papathanasiou et al. (2015)
TCF1	Transcription factor 1	Castano Betancourt et al. (2012)
TCF4	Transcription factor 4	Castano Betancourt et al. (2012) and Papathanasiou et al. (2012)
WISP	WNT1-inducible-signalhg pathway protein 1	van den Bosch et al. (2014)
WNT3α	Wingless-related integrationsite 3a	Leijten et al. (2013) and van den Bosch et al. (2014)
WNT8	Wingless-related integrated site 8	van den Bosch et al. (2014)
H2AX	Gamma-H2A histone family, member X	Guidotti et al. (2015)
	β-catenin	Borzi et al. (2010), Facchini et al. (2012), Papathanasiou et al. (2012), Chen et al. (2016)

**B: Ihh/PTHrP**		
EZH2	Enhancer of zeste homolog 2	Chen et al. (2016)
HES1	Hairy and enhancerof split-1	Hosaka et al. (2013) and Lin et al. (2016)
IHH	Indian hedgehog homolog	Chang et al. (2009), Saito et al. (2010), Wei et al. (2012), Zhou et al. (2014), Garciadiego-Cazares et al. (2015), Thompson et al. (2015), Chen et al. (2016), Yahara et al. (2016), Zhou et al. (2016), and Zhang et al. (2017)
Mef2c	Myocyte-specific enhancer factor 2C	Yahara et al. (2016)
mTOR	Mechanistic target of rapamycin	Zhang et al. (2017)
OC	Osteocalcin	Castano Betancourt et al. (2012), Cavaco et al. (2016), and Lin et al. (2016)
PAI1	Plasminogen-activator inhibitor-1	Ailixiding et al. (2015)
PPR/PTHR1	PTH-related protein receptor/parathyroid hormone 1 receptor	Zhang et al. (2017)
p-S6	Phospho-S6	Zhang et al. (2017)
PTCH1	Protein patched homolog 1	Thompson et al. (2015), and Lin et al. (2016)
PTCH2	Protein patched homolog 2	Zhou et al. (2014) and Lin et al. (2016)
PTHrP	Parathyroid hormone-related protein	Brew et al. (2010), Eswaramoorthy et al. (2012), Pesesse et al. (2014), and Zhang et al. (2017)
SIK3	Salt-lnducible kinase 3	Yahara et al. (2016)

**C: TGF-β superfamily**		
SOST	Sclerostin	Papathanasiou et al. (2015)
ALK1	Activin receptor-like kinase 1	Blaney Davidson et al. (2009) and van den Bosch et al. (2014)
ALK5	Activin receptor-like kinase 5	Blaney Davidson et al. (2009) and van den Bosch et al. (2014)
ATF2	Activating transcription factor 2	Li et al. (2010)
BAPX1/NKX3.2	Bagpipe homeobox homologl/NK3 homeobox 2	Chang et al. (2009) and Caron et al. (2015)
BMP-2	Bone morphogenetic protein 2	Papathanasiou et al. (2012)
BMP-4	Bone morphogenetic protein 4	Papathanasiou et al. (2012)
BMP-7	Bone morphogenetic protein 7	Papathanasiou et al. (2012) and Garciadiego-Cazares et al. (2015)
BMPR1A	Bone morphogenetic protein receptor, type 1A	Papathanasiou et al. (2012)
CAGA12 promotor activity		Gao et al. (2012)
FN	Fibronectin	Garciadiego-Cazares et al. (2015)
GDF-5	Growth/differentiation factor 5	Garciadiego-Cazares et al. (2015)
GREM1	Gremlin 1	Leijten et al. (2013)
	Integrin a1	Johnson et al. (2008) and Garciadiego-Cazares et al. (2015)
	Integrin a5	Garciadiego-Cazares et al. (2015)
	Integrin aV	Garciadiego-Cazares et al. (2015)
	Integrin b1	Garciadiego-Cazares et al. (2015)
MATN3	Matrilin 3	Yang et al. (2014)
SMAD1	Mothers against decapentaplegic homolog 1	Gao et al. (2012), Yang et al. (2014), and Papathanasiou et al. (2015)
SMAD2	Mothers against decapentaplegic homolog 2	Gao et al. (2012)
SMAD3	Mothers against decapentaplegic homolog 3	Li et al. (2010) and Gao et al. (2012)
SMAD5	Mothers against decapentaplegic homolog 5	Gao et al. (2012) and Papathanasiou et al. (2015)
SMAD8	Mothers against decapentaplegic homolog 8	Gao et al. (2012) and Papathanasiou et al. (2015)
TGF-β	Transforming growth factor beta	van den Bosch et al. (2014)

**D: MAPK/ERK**		
(p)ERK	(phosphorylated) extra-cellular-regulated kinases	Prasadam et al. (2010), Prasadam et al. (2013), Bianchi et al. (2016), Xu et al. (2016), and Zhang et al. (2017)
(p)JNK	(phosphorylated) c-Jun N-terminal kinase	Xu et al. (2016)
(p-)P38	(phosphorylated) P38	Johnson et al. (2008), Prasadam et al. (2013), Philipot et al. (2014), Bianchi et al. (2016), and Xu et al. (2016)
FXIIIA, F13A	Factor XIII	Johnson et al. (2008)
	Integrin α1β1	Garciadiego-Cazares et al. (2015)
(p)FAK	(phosphorylated) focal adhesion kinase	Johnson et al. (2008)
TG2, TGM2	Transglutaminase 2	Johnson et al. (2008) and Huebner et al. (2009)

**E: Inflammatory signaling**		
AP-2 ε	Activating enhancer binding protein 2 epsilon	Wenke et al. (2009) and Wenke et al. (2011)
CD36	Cluster of differentiation 36	Cecil et al. (2009)
CD45	Cluster of differentiation 45	Cavaco et al. (2016)
COX2	Cyclo-oxygenase 2	Caron et al. (2015) and Cavaco et al. (2016)
CXCL1	Chemokine (C-X-C motif) ligand 1	Wenke et al. (2011)
CXCL6	Chemokine (C-X-C motif) ligand 6	Sherwood et al. (2015)
CXCR2	C-X-C motif chemokine receptor 2	Sherwood et al. (2015)
HDAC2	Histone deacetylase 2	Queirolo et al. (2016)
HDAC4	Histone deacetylase 4	Lu et al. (2014a) and Queirolo et al. (2016)
IKKα	Inhibitor of nuclear factor kappa-B kinase subunit alpha	Olivotto et al. (2008) and Guidotti et al. (2015)
IKKβ	Inhibitor of nuclear factor kappa-B kinase subunit beta	Olivotto et al. (2008) and Guidotti et al. (2015)
IL-1β	lnterleukin-1β	Thompson et al. (2015), Cavaco et al. (2016) and Nasi et al. (2016)
IL-6	lnterleukin-6	Philipot et al. (2014), Ailixiding et al. (2015), Caron et al. (2015), and Nasi et al. (2016)
IL-8	Interleukin-8	Pesesse et al. (2014) and Philipot et al. (2014)
iNOS	Nitric oxide synthase	Aini et al., 2012
LOX-1	Lectin-like oxidized low-density lipoprotein receptor-1	Hashimoto et al. (2016)
MiR24	MicroRNA24	Philipot et al. (2014)
MiR320	MicroRNA320	Meng et al. (2016)
NF-kB	Nuclear factor kappa-light-chain-enhancer of activated Bcells	Ijiri et al. (2008)
NITEGE	Aggrecan neoepitopes	Cecil et al. (2009)
ODC	Ornithine decarboxylase	Facchini et al. (2012)
PGE2	Prostaglandin E2	Caron et al. (2015) and Cavaco et al. (2016)
PKCε	Protein kinase C epsilon type	Queirolo et al. (2016)
	P16INK4a	Philipot et al. (2014)
RAGE	Receptor for advanced glycation end products	Cecil et al. (2009)
S100A11	S100 calcium-binding protein A11	Cecil et al. (2009)
TNFα	Tumor necrosis factor alpha	Lai et al. (2014) and Ailixiding et al. (2015)
SIRT-1	Sirtuin-1	Fujita et al. (2011)
SIRT-6	Sirtuin-6	Ailixiding et al. (2015)

**F: Hypoxic and angiogenic factors**		
BSP/OPN	Bone sialoprotein/osteopontin	Fukai et al. (2010), Pesesse et al. (2013), Pesesse et al. (2014), Cavaco et al. (2016), and Staines et al. (2016)
C/EBPβ	CCAAT-enhancer-binding proteins	Hirata et al. (2012)
CHM-1	Chondromodulin-1	Wang et al. (2012) and Zhang et al. (2016a,b)
CTGF	Connective tissue growth factor	Wang et al. (2012)
DDR2	Discoid in domain receptor 2	Zhang et al. (2014a,b)
DIO2	Type II iodothyronine deiodinase	Bomer et al. (2015)
HAS2	Hyaluronan synthase 2	Markway et al. (2013)
HIF-1α	Hypoxia-inducible factor 1α	Markway et al. (2013) and Zhang et al. (2016b)
HIF-2α/EPAS1	Hypoxia-inducible factor 2α/	Saito et al. (2010), Markway et al. (2013), Bomer et al. (2015), and Zhang et al. (2016b)
	endothelial PAS domain-containing	
	protein 1	
HIF-3α	Hypoxia-inducible factor 3α	Markway et al. (2015)
	Hypoxia	Markway et al. (2013, 2015)
TSP	Thrombospondin-1	Pesesse et al. (2014)
VEGF	Vascular endothelial growth factor	Johnson et al. (2008), Ray and Ray (2008), Borzi et al. (2010), Brew et al. (2010), Fukai et al. (2010), Saito et al. (2010), Hirata et al. (2012), Wang et al. (2012), Hosaka et al. (2013), Bianchi et al. (2016), and Zhang et al. (2016a,b)

**G: FGF**		
FGF23	Fibroblast growth factor 23	Orfanidou et al. (2009) and Bianchi et al. (2016)
FGFR1	Fibroblast growth factor receptor 1	Bianchi et al. (2016)
FGFR2	Fibroblast growth factor receptor 2	Bianchi et al. (2016)
FGFR3	Fibroblast growth factor receptor 3	Bianchi et al. (2016), Zhou et al. (2016), and Zhang et al. (2017)
FGFR4	Fibroblast growth factor receptor 4	Bianchi et al. (2016)
	Klotho	Bianchi et al. (2016)
mTOR	Mechanistic target of rapamycin	Zhang et al. (2017)
	P73	Zhang et al. (2017)
p-36	Phospho-36	Zhang et al. (2017)

**H: Notch**		
GLI1	GLI family zinc finger 1	Thompson et al. (2015) and Lin et al. (2016)
GLI2	GLI family zinc finger 2	Thompson et al. (2015) and Lin et al. (2016)
Hes1	Hairy and enhancer of split-1	Hosaka et al. (2013) and Lin et al. (2016)
JAG1	Jagged 1	Lin et al. (2016)
NICD1	Notch intracellular domain 1	Hosaka et al. (2013) and Lin et al. (2016)
NICD2	Notch intracellular domain 2	Hosaka et al. (2013)
NOTCH		Hosaka et al. (2013) and Lin et al. (2016)
RBPjκ	Recombination signal binding protein for immunoglobulin kappa J	Hosaka et al. (2013)

**I: Mineralization**		
ANK	Progressive ankylosis protein	Nguyen et al. (2013) and Nasi et al. (2016)
AKT1	RAC-alphaserine/threonine-protein kinase	Sherwood et al. (2015) and Bianchi et al. (2016)
Anx5	Annexin 5	Nasi et al. (2016)
BCP	Basic calcium phosphate	Fuerst et al. (2009) and Nasi et al. (2016)
CA	Carbonated-apatite	Nasi et al. (2016)
Ca^2+^	Calcium	Olivotto et al. (2008), Fuerst et al. (2009), Facchini et al. (2012), Nguyen et al. (2013), Olivotto et al. (2013), Cavaco et al. (2016), Nasi et al. (2016), Queirolo et al. (2016), and Yahara et al. (2016)
cOMP	Cartilage oligomeric matrix protein	Lai et al. (2014) and Cavaco et al. (2016)
CPPD	Calcium pyrophosphate dehydrates	Nasi et al. (2016)
	Fetuin	Wallin et al. (2010)
GGCX	Vitamin K-dependent gamma-carboxylase	Cavaco et al. (2016)
[(un)carboxylated] GRP	Gla-rich protein	Cavaco et al. (2016)
HA	Hydroxyapatite	Nasi et al. (2016)
IL-6	interleukin-6	Nasi et al. (2016)
[(un)carboxylated] MGP	Matrix Gla protein	Wallin et al. (2010) and Cavaco et al. (2016)
NTPPPH	Nucleoside triphosphate pyrophosphohydrolase	Pesesse et al. (2013)
OCP	Octacalcium phosphate	Nasi et al. (2016)
OCRL1	Lowe oculocerebrorenal syndrome protein	Zhu et al. (2015)
osx	Osterix	Cavaco et al. (2016)
PC-1/ENPP1, NPP1	Plasma-cell membrane glycoprotein 1/ectonucleotide pyrophosphatase/phosphodiesterase 1	Nguyen et al. (2013) and Nasi et al. (2016)
Pi	inorganic phosphate	Fukai et al. (2010)
PIT1	Inorganic phosphate transporter 1	Nguyen et al. (2013) and Nasi et al. (2016)
PIT2	Inorganic phosphate transporter 2	Nasi et al. (2016)
PKCε	Protein kinase C epsilon type	Queirolo et al. (2016)
Ppi	Inorganic pyrophosphate	Fukai et al. (2010)
RAC1	Ras-related C3 botulinum toxin substrate 1	Wang and Beier (2005)
TNAP	Transporter and tissue-nonspecific alkaline phosphatases	Nguyen et al. (2013) and Nasi et al. (2016)
VKOR	Vitamin K epoxide reductase	Cavaco et al. (2016)
	y-Carboxylase activity	Wallin et al. (2010)

**J: Hypertrophic differentiation markers**		
AGC, ACAN	Aggrecan	Chang et al. (2009), Aini et al., 2012, Castano Betancourt et al. (2012), Papathanasiou et al. (2012), Markway et al. (2013), Lai et al. (2014), Lu et al. (2014a), Philipot et al. (2014), Ailixiding et al. (2015), Bomer et al. (2015), Caron et al. (2015), Garciadiego-Cazares et al. (2015), Sherwood et al. (2015), Filip et al. (2016), Xu et al. (2016), and Zhang et al. (2016a,b)
ADAMTS1	A disintegrin and metalloproteinase with thrombospondin motifs 1	Lai et al. (2014)
ADAMTS4	A disintegrin and metalloproteinase with thrombospondin motifs 4	Little et al. (2009), Lai et al. (2014), Lu et al. (2014a), and Chen et al. (2016)
ADAMTS5	A disintegrin and metalloproteinase with thrombospondin motifs 5	Huebner et al. (2009), Hirata et al. (2012), Prasadam et al. (2013), Lai et al. (2014), Lu et al. (2014a), Bomer et al. (2015), Caron et al. (2015), Thompson et al. (2015), Zhu et al. (2015), Chen et al. (2016), Lin et al. (2016), Nasi et al. (2016), Xu et al. (2016), and Zhou et al. (2016)
	
ADAMTS7	A disintegrin and metalloproteinase with thrombospondin motifs 7	Lai et al. (2014)
ALPL	Alkaline phosphatase	Chang et al. (2009), Prasadam et al. (2010), Hirata et al. (2012), Pesesse et al. (2013), Zhang et al. (2014a,b), Bomer et al. (2015), Caron et al. (2015), Zhu et al. (2015), Filip et al. (2016), Yahara et al. (2016), and Zhang et al. (2016a)
CASP3	Caspase3	Xu et al. (2016)
COL1	Type 1 collagen	Castano Betancourt et al. (2012), Gao et al. (2012), Markway et al. (2013), Nagase et al. (2013), Bomer et al. (2015), Bianchi et al. (2016), and Yahara et al. (2016)
COL10A1	Type X collagen	Johnson et al. (2008), Cecil et al. (2009), Chang et al. (2009), Huebner et al. (2009), Wu et al. (2009), Borzi et al. (2010), Brew et al. (2010), Fukai et al. (2010), Li et al. (2010), Prasadam et al. (2010), Saito et al. (2010), Fujita et al. (2011), Aini et al., 2012, Castano Betancourt et al. (2012), Eswaramoorthy et al. (2012), Facchini et al. (2012), Gao et al. (2012), Hirata et al. (2012), Papathanasiou et al. (2012), Wei et al. (2012), Hosaka et al. (2013), Markway et al. (2013), Olivotto et al. (2013), Pesesse et al. (2013), Prasadam et al. (2013), Lai et al. (2014), Lu et al. (2014b), Pesesse et al. (2014), Yang et al. (2014), Zhang et al. (2014b), Zhou et al. (2014), Ailixiding et al. (2015), Bomer et al. (2015), Caron et al. (2015), Markway et al. (2015), Sherwood et al. (2015), Zhu et al. (2015), Bianchi et al. (2016), Cavaco et al. (2016), Chen et al. (2016), Filip et al. (2016), Hashimoto et al. (2016), Lin et al. (2016), Nasi et al. (2016), Queirolo et al. (2016), Staines et al. (2016), Yahara et al. (2016), Zhang et al. (2016a), Zhang et al. (2016b), Zhou et al. (2016), and Zhang et al. (2017)
COL2A1	Type II collagen	Johnson et al. (2008), Chang et al. (2009), Huebner et al. (2009), Wu et al. (2009), Aini et al., 2012, Castano Betancourt et al. (2012), Eswaramoorthy et al. (2012), Facchini et al. (2012), Gao et al. (2012), Papathanasiou et al. (2012), Markway et al. (2013), Nagase et al. (2013), Olivotto et al. (2013), Prasadam et al. (2013), Lu et al. (2014a), Zhang et al. (2014b), Zhou et al. (2014), Bomer et al. (2015), Caron et al. (2015), Garciadiego-Cazares et al. (2015), Markway et al. (2015), Sherwood et al. (2015), Bianchi et al. (2016), Cavaco et al. (2016), Filip et al. (2016), Nasi et al. (2016), Queirolo et al. (2016), Xu et al. (2016), Yahara et al. (2016), and Zhang et al. (2016a)
COL3	Type III Collagen	Gao et al. (2012) and Bianchi et al. (2016)
CTS	Cathepsin	Appleton et al. (2007) and Zhu et al. (2015)
GAG	Glycosaminoglycans	Chang et al. (2009), Markway et al. (2013), Bomer et al. (2015), Garciadiego-Cazares et al. (2015), and Sherwood et al. (2015)
MMP1	Matrix metalloproteinase 1	Ray and Ray (2008), Wei et al. (2012), Markway et al. (2013), Lai et al. (2014), Lu et al. (2014a), Philipot et al. (2014)
MMP10	Matrix metalloproteinase 10	Olivotto et al. (2013) and Guidotti et al. (2015)
	Matrix metalloproteinase 13	Appleton et al. (2007), Johnson et al. (2008), Blaney Davidson et al. (2009), Huebner et al. (2009), Orfanidou et al. (2009), Borzi et al. (2010), Brew et al. (2010), Saito et al. (2010), Facchini et al. (2012), Hirata et al. (2012), Papathanasiou et al. (2012), Wei et al. (2012), Hosaka et al. (2013), Markway et al. (2013), Nagase et al. (2013), Olivotto et al. (2013), Pesesse et al. (2013), Lai et al. (2014), Lu et al. (2014a), Philipot et al. (2014), Zhang et al. (2014b), Zhou et al. (2014), Ailixiding et al. (2015), Bomer et al. (2015), Caron et al. (2015), Markway et al. (2015), Thompson et al. (2015), Bianchi et al. (2016), Cavaco et al. (2016), Chen et al. (2016), Filip et al. (2016), Meng et al. (2016), Nasi et al. (2016), Queirolo et al. (2016), Staines et al. (2016), Xu et al. (2016), Zhang et al. (2016a), Zhang et al. (2016b), and Zhou et al. (2016)
MMP14	Matrix metalloproteinase 14	Markway et al. (2013) and Lai et al. (2014)
MMP2	Matrix metalloproteinase 2	Markway et al. (2013) and Prasadam et al. (2013)
MMP3	Matrix metalloproteinase 3	Hirata et al. (2012), Wei et al. (2012), Markway et al. (2013), Lai et al. (2014), Lu et al. (2014a), Chen et al. (2016), and Nasi et al. (2016)
MMP9	Matrix metalloproteinase 9	Ray and Ray (2008), Hirata et al. (2012), Wang et al. (2012), Nagase et al. (2013), and Lai et al. (2014)
	Osteophyte	Ray and Ray (2008), Fukai et al. (2010), and Lin et al. (2016)
PCNA	Proliferating cell nuclear antigen	Zhou et al. (2016)
PRG4	Proteoglycan 4/lubricin	Yahara et al. (2016), Zhou et al. (2016)
	Proteoglycans	Hirata et al. (2012), Wei et al. (2012), Hosaka et al. (2013), Prasadam et al. (2013), Lin et al. (2016), Queirolo et al. (2016), Yahara et al. (2016), Aini et al., 2012, Eswaramoorthy et al. (2012), Ailixiding et al. (2015), Caron et al. (2015), Markway et al. (2015), Bianchi et al. (2016), Filip et al. (2016), Hashimoto et al. (2016), Lin et al. (2016), Nasi et al. (2016), Xu et al. (2016), Yahara et al. (2016), and Zhang et al. (2016a,b)
RUNX2/CBFα1	Runt-related transcription factor 2/Core-binding factor subunit alpha-1	Olivotto et al. (2008), Orfanidou et al. (2009), Borzi et al. (2010), Prasadam et al. (2010), Saito et al. (2010), Facchini et al. (2012), Hirata et al. (2012), Olivotto et al. (2013), Pesesse et al. (2013), Lu et al. (2014b), Zhou et al. (2014), Bomer et al. (2015), Caron et al. (2015), Zhu et al. (2015), Bianchi et al. (2016), Hashimoto et al. (2016), Nasi et al. (2016), Queirolo et al. (2016), Yahara et al. (2016), Zhang et al. (2016a,b), Zhou et al. (2016), and Zhang et al. (2017)
	
SOX9		Olivotto et al. (2008), Chang et al. (2009), Orfanidou et al. (2009), Wenke et al. (2009), Borzi et al. (2010), Facchini et al. (2012), Lu et al. (2014b), Caron et al. (2015), Sherwood et al. (2015), Bianchi et al. (2016), Chen et al. (2016), Filip et al. (2016), Nasi et al. (2016), Queirolo et al. (2016), Yahara et al. (2016), and Zhang et al. (2016a)

**K: Not pathway associated**		
	Bone bridges	Staines et al. (2016)
	Bone volume/density	Lu et al. (2014b)
	Cell adhesion	Pesesse et al. (2013)
	Cell number	Olivotto et al. (2008)
	Cell size	Olivotto et al. (2008)
	Cortical bone	Lu et al. (2014b)
	Femur length	Lu et al. (2014b)
	Hypertrophic cells	Pesesse et al. (2013)
	Proliferation	Guidotti et al. (2015), Staines et al. (2016)
	Thickness	Prasadam et al. (2013), Zhou et al. (2016)
	Total cartilage area	Zhang et al. (2017)
	Wound healing	Pesesse et al. (2013)
ACTA1	Actin, Alpha 1	Appleton et al. (2007)
AQP1	Aquaporin 1	Nagase et al. (2013)
ASPN	Asporin	Nagase et al. (2013)
BST1	Bone marrow stromal cell antigen-1	Appleton et al. (2007)
CIS	Complement C1s	Appleton et al. (2007)
CASQ2	Calsequestrin-2	Appleton et al. (2007)
CD14	Cluster of differentiation 14	Appleton et al. (2007)
CD53	Cluster of differentiation 53	Appleton et al. (2007)
CHI3L1	Chitinase-3 like 1	Appleton et al. (2007)
CHN2	Chimerin 2	Appleton et al. (2007)
CXCR4	C-X-C motif chemokine receptor 4	Appleton et al. (2007)
CYBB/Nox2	Cytochrome b-245 heavy chain/NADPH oxidase 2	Appleton et al. (2007)
CYP4B1	Cytochrome P450 4B1	Nagase et al. (2013)
DBP	D site of albumin promoter binding protein	Appleton et al. (2007)
DCAMKL1	Doublecortin-like kinase 1	Appleton et al. (2007)
ECM1	Extracellular Matrix Protein 1	Appleton et al. (2007)
F3	Coagulation Factor IIl	Appleton et al. (2007)
FCGR3	Low affinity immunoglobulin gamma Fc region receptor III-A	Appleton et al. (2007)
GADD5A	Growth arrest and DNA-damage-inducible 45 alpha protein	Appleton et al. (2007)
GAP43	Growth-associated protein 43	Appleton et al. (2007)
GAS-6	Growth arrest-specific 6	Appleton et al. (2007)
GBP2	Guanylate binding protein 2	Appleton et al. (2007)
GPM6b	Glycoprotein M6B	Appleton et al. (2007)
HFE	Human hemochromatosis protein	Appleton et al. (2007)
IGFbp6	Insulin like growth factor binding protein 6	Appleton et al. (2007)
IGSF6	Immunoglobulin superfamily member 6	Appleton et al. (2007)
IL2RG	lnterteukin-2 receptor subunit gamma	Appleton et al. (2007)
LBP	Lipopolysaccharide binding protein	Appleton et al. (2007)
LTBP2	Latent transforming growth factor beta binding protein 2	Appleton et al. (2007)
MCAM	Melanoma cell adhesion molecule	Appleton et al. (2007)
MGL	Monoacylglycerol lipase	Appleton et al. (2007)
MPEG1	Macrophage expressed 1	Appleton et al. (2007)
MT1A	Metallothionein 1A,	Appleton et al. (2007)
NR1D1	Nuclear receptor subfamily 1 Group D Member 1	Appleton et al. (2007)
PER3	Period circadian protein homolog 3	Appleton et al. (2007)
PTPRC	Protein tyrosine phosphatase, receptor type C	Appleton et al. (2007)
PTPRO	Protein tyrosine phosphatase, receptor type O	Appleton et al. (2007)
RELN	Reelin	Appleton et al. (2007)
RGS5	Regulator of G protein signaling 5	Appleton et al. (2007)
SCNN1A	Sodium channel epithelial 1 alpha subunit	Nagase et al. (2013)
SERPIN1	Serine protease inhibitor 1	Appleton et al. (2007)
SPON1	Spondin 1	Nagase et al. (2013)
THBD	Thrombomodulin	Appleton et al. (2007)
THBS4	Thrombospondin-4	Appleton et al. (2007)
TLR2	Toll-like receptor 2	Appleton et al. (2007)

*After our literature search, the remaining 76 papers reporting on chondrocyte hypertrophy in osteoarthritis (OA) were screened for markers which were reported by the authors to be associated with the phenotypic change in OA. Markers are ordered by their associated signaling pathway or other groups*.

It is well established that β-catenin signaling is associated with chondrocyte hypertrophy (Wu et al., 2009; Borzi et al., 2010; Castano Betancourt et al., 2012; Facchini et al., 2012; Papathanasiou et al., 2012; Leijten et al., 2013; van den Bosch et al., 2014; Guidotti et al., 2015; Chen et al., 2016; Staines et al., 2016). Next to its role in chondrocyte hypertrophy, recent advancements in knowledge regarding OA and the involvement of Wnt signaling now demonstrate that expression levels of Wnt signaling- and β-catenin-inducing factors, as well as downstream Wnt effectors, such as LEF1 and AXIN2, are directly or indirectly associated with OA (Wu et al., 2009; Borzi et al., 2010; Castano Betancourt et al., 2012; Facchini et al., 2012; Papathanasiou et al., 2012; Leijten et al., 2013; van den Bosch et al., 2014; Guidotti et al., 2015; Chen et al., 2016; Staines et al., 2016).

A causative relationship between canonical Wnt signaling and OA initiation/progression has been suggested by two recent studies. Cartilage-specific SMURF2-mediated ubiquitination and proteasomal degradation of GSK-3β resulted in increased β-catenin signaling (Wu et al., 2009). Tibial and femoral articular cartilage in this *Col2a1-Smurf2* mouse model demonstrated that 2.5-week-old mice displayed an increased basal layer of the deep articular cartilage with higher *Col10a1* expression. These early hypertrophic changes in the articular cartilage of these mice were subsequently followed by cartilage degeneration and osteophyte formation when the mice became older (Wu et al., 2009), suggesting a direct relationship between early hypertrophic changes followed by OA development. Another study by Chen et al. provided evidence for the activation of Wnt/β-catenin signaling in OA development. This study investigated the effects of EZH2 inhibition on OA development in a surgically induced OA mouse model (Chen et al., 2016). EZH2 is the catalytic unit of the polycomb repressive complex 2 (PRC2), responsible for transcriptional silencing of a multitude of genes involved in differentiation (Morey and Helin, 2010). EZH2 expression was higher in OA chondrocytes compared to healthy chondrocytes and overexpression of EZH2 in normal chondrocytes resulted in activation of β-catenin signaling, including higher mRNA expression of its downstream effectors, *AXIN2* and *LEF1*. Confirming the association between increased β-catenin signaling and OA development, intra-articular injection with a pharmacological EZH2 inhibitor in a surgically induced mouse OA model resulted in reduced cartilage degradation compared to mice injected with a saline control. In the cartilage this was accompanied by reduced mRNA expression of *Col10a1, Adamts5, Mmp13, Mmp3*, and increased mRNA expression of the Wnt inhibitor *Sfrp1* as well as lower mRNA expression of β-catenin.

Other studies provide additional links between the Wnt/β-catenin pathway and OA (Borzi et al., 2010; Castano Betancourt et al., 2012; Papathanasiou et al., 2012; Leijten et al., 2013; van den Bosch et al., 2014). The expression of Wnt and BMP antagonists dickkopf 1 homolog (*DKK1*), frizzled-related protein (*FRZB*), and Gremlin 1 (*GREM1*) were reduced in OA cartilage compared to post-mortem healthy controls (Leijten et al., 2013). These Wnt and BMP antagonists were able to inhibit hypertrophic chondrocyte differentiation when added to chondrogenically differentiated mesenchymal stem cells (MSCs). This study also described crosstalk between the Wnt pathway and BMP signaling pathway (Leijten et al., 2013). This was functionally revealed through the decreased expression of the Wnt target gene *AXIN2* (axis inhibition protein 2) and the BMP target gene *ID1* (DNA-binding inhibitor protein 1), following treatment with BMP-2 or WNT3A, respectively (Leijten et al., 2013). This feedback loop allows tight control and balance between BMP and Wnt signaling (Leijten et al., 2013). New insight into this crosstalk between Wnt and BMP signaling pathways was obtained through the observation that BMP-2-induced Wnt signaling through influencing the SMAD1/5/8-depending LRP5 promoter activity in human OA articular chondrocytes (Papathanasiou et al., 2012). LRP5 is a co-receptor of the Wnt/β-catenin signaling pathway and its expression is increased in OA chondrocytes (Papathanasiou et al., 2012). It was indicated that the increase in mRNA levels of genes, such as *Col10a1, Mmp13*, and *Adamts5* after BMP stimulation could be abrogated by LRP5 siRNA-mediated knockdown, indicating that the hypertrophic effects of BMP signaling may promote cartilage destruction *via* increased Wnt/β-catenin signaling (Papathanasiou et al., 2012). Other intriguing crosstalk was identified between Wnt and TGF-β signaling through the finding that downstream TGF-β activity is altered after chondrocytes were exposed to WNT3A and the downstream canonical Wnt signaling protein WISP1 (van den Bosch et al., 2014). *In vitro* stimulation of chondrocytes or *in vivo* viral expression of WNT3, WNT8, and or WISP1 skews TGF-β signaling from ALK5 (resulting in SMAD 2/3 signaling), toward signaling *via* ALK1 (resulting in SMAD 1/5/8 phosphorylation), inducing a hypertrophic chondrocyte phenotype (van den Bosch et al., 2014).

Further support associating Wnt signaling to OA development comes from a study in which a single nucleotide polymorphism in the *DOT1L* gene results in a reduced risk for hip OA. The underlying mechanism was identified as decreased Wnt signaling activity, which was confirmed *via* reduced expression levels of the Wnt target genes, *AXIN2* and *TCF1*. DOT1L was found to be involved in chondrogenic differentiation and is thought to co-transcriptionally regulate transcription of Wnt-target genes *via* direct interaction with TCF4 (Castano Betancourt et al., 2012). Activation of Wnt signaling *via* LiCl-mediated GSK3-β inactivation led to chondrocyte cellular senescence, as indicated by increased *p21* expression, production of reactive oxygen species, SAβ galactosidase activity, and activation of the DNA damage response (Guidotti et al., 2015). Moreover it was concluded that the inhibition of GSK3-β activity promotes a chondrocyte hypertrophic phenotype, thereby supporting that Wnt signaling activity has an important balancing influence on major cell biological parameters with consequences for the chondrocyte phenotype (Guidotti et al., 2015). ShRNA-mediated MMP13 knockdown in primary human chondrocytes resulted in reduced expression of β-catenin. This was accompanied by reduced expression of RUNX2, and an increased nuclear presence of SOX9 as well as a higher glycosaminoglycan (GAG) content. This indicates that a loss of MMP13 may ameliorate chondrocyte homeostasis in a feedback loop *via* reduction of β-catenin levels (Borzi et al., 2010).

In another study, it was suggested that increased Wnt signaling may be associated with OA progression (Staines et al., 2016). STR/Ort mice develop OA spontaneously, and expression of the Wnt signaling inhibitor sclerostin (*Sost*) decreased during OA progression in these mice. Furthermore, sclerostin expression was reduced in regions with more cartilage degradation, again linking increased Wnt signaling to cartilage degradation. Intriguingly, this study also provided links between endochondral growth defects and OA progression. STR/Ort mice display an abnormal growth plate development, with greater expression levels of COL10A1 and MMP13. This supports an association between endochondral defects and cartilage degradation (Staines et al., 2016). In contrast to the study by Staines *et al*. Papathanasiou et al. found an increased expression of the Wnt inhibitor sclerostin in human OA chondrocytes compared to normal (Papathanasiou et al., 2015). This was accompanied with a decreased methylation of the *SOST* promotor, enhancing the binding affinity of SMAD1/5/8 to the CpG region of the *SOST* promotor. Whether upregulation of *SOST* expression in articular chondrocytes is a causal factor or a result in OA has to be further determined (Papathanasiou et al., 2015).

Overall these studies together confirm the involvement of canonical Wnt signaling in chondrocyte hypertrophy and revealed novel mechanisms that appear to tune canonical Wnt signaling responses and which relate to the development of OA.

### Ihh/PTHrP Signaling

**Figure 3:**
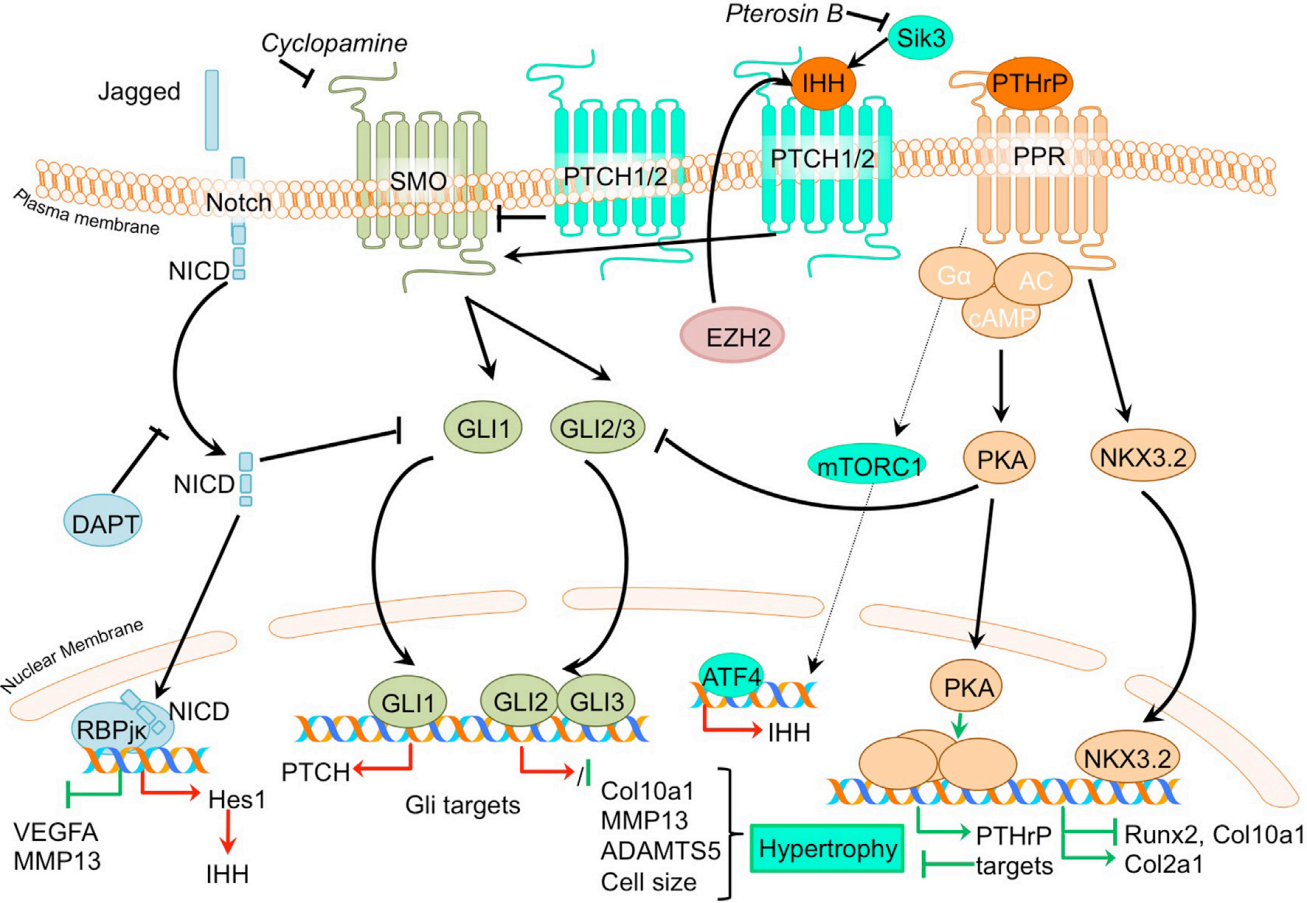
Overview of the canonical Ihh/PTHrP and Notch signaling pathways and newly acquired insights into their relation to the development of the osteoarthritic (OA) chondrocyte hypertrophic phenotype. The canonical pathway was updated with recent insights in the role of Ihh/PTHrP and Notch signaling in the hypertrophic switch occuring during OA development. In green pro-chondrogenic relations are shown, while pro-hypertrophic relations are depicted in red.

PTHrP- and Indian hedgehog signaling pathways generate a feedback loop which is involved in controlling the chondrocyte phenotype in the growth plate in skeletal development, as well as in determining the homeostasis that keeps articular cartilage healthy (Kronenberg, 2003). When IHH reaches its target cell, it binds to the Patched-1 (PTCH1) receptor. In the absence of ligand, PTCH1 inhibits Smoothened (SMO), the binding of IHH relieves SMO inhibition, leading to activation of the GLI transcription factors: the activators GLI1 and GLI2 and the repressor GLI3. Activated GLI accumulates in the nucleus and controls the transcription of hedgehog target genes (Yang et al., 2015). Insight into the mechanism of action of PTH and PTHrP has been provided by the discovery of the type I PTH/PTHrP receptor (PTHR). This G protein-coupled receptor (GPCR) is associated with, among others, the adenylyl cyclase/protein kinase A (PKA) pathway (Mannstadt et al., 1999). The PTHrP/Ihh feedback loop is a major determinant of the chondrocyte hypertrophic phenotype, and thus postulated to be pivotal in OA development. It is thought that part of the contra-hypertrophic action of PTHrP originates from inducing the expression of BAPX1/NKX3.2 (Provot et al., 2006), a potent hypertrophic switch (Caron et al., 2015). Some of the pertinent evidence that chondrocyte hypertrophy is an intrinsic part of OA development comes from papers regarding PTHrP/Ihh signaling. An overview of the newly acquired insights into this pathway and its involvement in development of OA chondrocyte hypertrophy is provided in Table 1b and Figure 3.

Given that PTHrP maintains the function of proliferating chondrocytes in the growth plate and inhibits chondrocyte differentiation toward hypertrophy, it has been suggested that PTHrP may be protective against OA (Kronenberg, 2003). Several studies demonstrate direct evidence that inhibiting hypertrophic processes results in protection against OA. Confirming an anti-hypertrophic and OA protective effect of PTHrP, it was shown that PTHrP can inhibit terminal differentiation of normal human articular chondrocytes and that intra-articular PTHrP administration in a chemically induced rat OA model reduces OA, as evidenced by reduced GAG loss (Chang et al., 2009). Along with reduced matrix loss, PTHrP treatment of chemically OA-induced knees also resulted in increased *Col2a1* levels and reduced *Col10a1* levels in the cartilage compared to OA-induced knees without PTHrP injection (Chang et al., 2009). Another study confirmed the chondroprotective and anti-hypertrophic role of PTHrP signaling in cartilage by revealing that incorporating PTHrP into a drug delivery system reduced OA severity in a chemically induced mouse OA model. This was evidenced through reduced GAG loss accompanied by decreased COL10A1 staining (Eswaramoorthy et al., 2012). The anti-hypertrophic role of PTHrP signaling was further confirmed in a study in which OA chondrocytes incorporated in alginate beads were treated with PTHrP, leading to reduced *COL10A1* expression (Pesesse et al., 2014).

While PTHrP has been associated with anti-hypertrophic actions in the growth plate and in OA, Ihh expression is considered as a marker gene for hypertrophic chondrocytes in growth plates (Weisser et al., 2002). In concert with an important role for Ihh in chondrocyte hypertrophy, inhibition of the Ihh pathway protected against OA development (Zhou et al., 2014). In this study, a cartilage-specific *Ihh* knockout mouse was used to genetically confirm that Ihh drives the development of OA (Zhou et al., 2014). Cartilage-specific deletion of *Ihh* largely protected against the development of post-traumatic OA. Next to the decreased OA severity [as determined by the OOCHAS score (Osteoarthritis Cartilage Histopathology Assessment System)], *Ihh*-deleted mice revealed decreased expression of COL10A1 and MMP13, as well as decreased activity of cartilage proteolytic enzymes. A study by Yahara et al. illustrated a reduction of hypertrophic changes in *Sik3* deficient mice including reduced *Ihh* levels (Yahara et al., 2016). The precise mechanism behind the anti-hypertrophic actions of Pterosin B *via* SIK3 is unknown, but KEGG (Kyoto Encyclopedia of Genes and Genomes) pathway analysis indicated an inhibition of *Ihh* by Pterosin B, which is a SIK3 inhibitor.

It was demonstrated that the expression of *IHH* is increased in OA cartilage compared to cartilage obtained from non-OA patients (Wei et al., 2012). Furthermore, *IHH* levels were also higher in OA synovial fluid compared to non-OA synovial fluid (Wei et al., 2012). Since, expression of *IHH* was higher in more severely degenerated cartilage areas, as determined by the modified Mankin score, this also indicated an association of cartilage hypertrophic changes with OA progression (Wei et al., 2012). The authors in this study provided important evidence that chondrocyte hypertrophy is involved in the association between *IHH* and OA, showing that OA chondrocytes treated with Ihh displayed increased mRNA expression levels of markers of chondrocyte hypertrophy *COL10A1* and *MMP13*. Furthermore, both *IHH* expression and chondrocyte size (the phenotypic hallmark of hypertrophy) were associated with OA severity. Modulation of Ihh signaling activity by the inhibitor cyclopamine caused a reduction of *COL10A1* and *MMP13* expression, suggesting that dampening the activity of the Ihh pathway may provide a target for OA treatment (Wei et al., 2012). Additional support connecting Ihh signaling to OA comes from an earlier mentioned study that described that the inhibition of EZH2 reduces cartilage degradation *in vivo*. It was found that *IHH* expression was increased after overexpression of *EZH2* in chondrocytes, and was associated with an overall increased hypertrophic phenotype (Chen et al., 2016).

To isolate the effects of hedgehog pathway activation, the consequences of its activation for cartilage degradation have been investigated (Thompson et al., 2015). In contrast to the large body of evidence for an OA promoting role of Ihh, studies in bovine and human cartilage explants revealed that activation of hedgehog signaling by exposure to IHH did not increase *ADAMTS5* or *MMP13* expression. Chondrocyte hypertrophic phenotype development was not extensively addressed in this study, but it appears that the catabolic effects of hedgehog signaling in OA may be context dependent.

Recent advancements in research regarding crosstalk with other signaling pathways shows that hedgehog signaling crosstalks with the Notch signaling pathway (Lin et al., 2016) and with the Wnt signaling, BMP signaling, FGF (Zhou et al., 2016), and mTOR signaling pathways (Leijten et al., 2013). Inhibition of Notch1 resulted in an increased, HES1 dependent, hedgehog signaling activity (Lin et al., 2016). This increased activity led to an exacerbation of experimental OA, characterized by increased levels hedgehog target genes, *osteocalcin, COL10A1, ADAMTS5*, osteophyte formation, and reduced OARSI scores (Lin et al., 2016). It is still not completely understood as to how Notch signaling controls hedgehog signaling. It has, however, been suggested that Notch signaling activity may limit hypertrophy-provoking hedgehog signaling in articular chondrocytes (Lin et al., 2016). Interestingly, mTORC1 activation in mouse articular cartilage from 2-month-old mice resulted in upregulation of Ihh expression, along with other hypertrophic markers, such as RUNX2 and COL10A1, suggesting interplay between mTOR and Ihh signaling (Zhang et al., 2017). Along with an early increase in hypertrophic markers, mice in which mTORC1 was activated developed progressive OA, together with an increasing ratio of calcified cartilage relative to hyaline cartilage through time. In this study, it was also suggested that mTORC1 activation inhibits PTHrP signaling *via* downregulating the PPR receptor which ultimately results in impaired prevention of the initiation of chondrocyte hypertrophic differentiation (Zhang et al., 2017). Ihh signaling was also shown to crosstalk with the fibroblast growth factor (FGF) pathway, since the OA-like changes in the temporomandibular joints of FGFR3 knockout mice could be ameliorated using an Ihh signaling inhibitor (Zhou et al., 2016).

Together these studies underline the involvement of the PTHrP/Ihh feedback loop in determining the chondrocyte phenotype, and recent developments reveal that a disruption of this well-balanced system can initiate a hypertrophic switch, ultimately leading toward OA disease initiation and progression.

### TGF-β Superfamily Signaling

The TGF-β superfamily is a group of multifunctional cytokines that play critical roles in cartilage homeostasis and have well established roles in endochondral ossification (Dangelo et al., 2001). In the classical TGF-β superfamily signaling pathway, superfamily ligands, such as TGF-β and BMP isoforms, bind to their respective cell surface receptors and, upon type I and -II receptor dimerization, activate a signaling cascade which includes the recruitment, phosphorylation, and interaction of different SMAD proteins. Upon activation, SMAD complexes translocate to the nucleus to drive transcription of genes regulating a variety of biological responses (van der Kraan et al., 2009). TGF-β has been strongly implicated in OA pathogenesis, since it has been well established that deregulation of TGF-β signaling is associated with OA (van der Kraan et al., 2009; van der Kraan, 2017). An overview of newly acquired insights into this pathway and its involvement in development of OA chondrocyte hypertrophy is provided in Table 1c and Figure 2.

Several studies have demonstrated an increased expression of TGF-β superfamily members or their receptors and down-stream signaling molecules in human OA chondrocytes compared to non-OA chondrocytes (Papathanasiou et al., 2012, 2015). TGF-β superfamily ligands can signal in chondrocytes *via* SMAD2/3 and also *via* SMAD1/5/8 (van der Kraan, 2017). The type I receptor ALK1 is associated with SMAD1/5/8 activity and hypertrophy and mineralization (Blaney Davidson et al., 2009). Additionally, a high correlation was found between ALK1 and the hypertrophic marker MMP13 (Blaney Davidson et al., 2009). In contrast, the type I receptor ALK5 is associated with SMAD2/3 activity and has anti-hypertrophic chondroprotective effects (van der Kraan et al., 2009). Together with the association of ALK1 with the hypertrophic marker MMP13, the study by Blaney Davidson et al. revealed that the ALK1/ALK5 ratio is increased in a post-traumatic model of OA using the destabilization of the medial meniscus (DMM) mouse OA model, and also increased with OA progression (Blaney Davidson et al., 2009), again highlighting the involvement of chondrocyte hypertrophic processes in OA development. The association of SMAD1/5/8 signaling with chondrocyte hypertrophy and OA is further confirmed in another study by Yang et al. Here, an association between the noncollagenous extracellular matrix protein Matrilin-3 (MATN3), and the SMAD1 pathway was confirmed (Yang et al., 2014). Authors of this study had earlier shown that *Matn3* KO mice displayed premature articular cartilage hypertrophy and accelerated OA-like joint pathology (van der Weyden et al., 2006) and sought to determine molecular explanations for increased chondrocyte hypertrophy after *Matn3* knockout. It was demonstrated that BMP-2 expression in embryonic chicken chondrocytes and a murine chondrocyte cell line lead to increased SMAD1 phosphorylation, resulting in increased *Col10a1* promotor activity (Yang et al., 2014). MATN3 was able to directly interact with BMP-2 and acts as a BMP-2 antagonist inhibiting BMP-2-induced SMAD1 phosphorylation and reducing *Col10a1* expression levels and chondrocyte hypertrophy.

TGF-β superfamily members can tune the activity and levels of chondrocyte phenotype-determining downstream transcriptional regulators. Examples of these downstream transcriptional regulators controlling the chondrocyte phenotype are BAPX1/NKX3.2 and ATF2. It was demonstrated that the anti-hypertrophic effect of BMP-7 on OA chondrocytes, evidenced by a reduction in mRNA expression levels of *COL10A1, MMP13*, and *RUNX2*, could be blocked with BAPX1/NKX3.2 knockdown, indicating that BMP-7 suppresses the hypertrophic phenotype in OA chondrocytes *via* BAPX1/NKX3.2 (Caron et al., 2015). ATF2 has been demonstrated to be expressed in the resting and proliferating zones of the growth plate, but not the hypertrophic zone (Reimold et al., 1996). It was established that ATF2 phosphorylation via TAK1 and p38 (which activates ATF2) was decreased as a consequence of SMAD3 ablation in DMM-induced OA in mice (Li et al., 2010). This study also revealed that ATF2 was able to reduce the increase in *Col10a1* mRNA expression levels induced by BMP-2 stimulation, hinting at ATF2 as a protective factor to dampen chondrocyte hypertrophy.

TGF-β superfamily members have also been revealed to modulate the expression of integrins, which are ECM receptors involved in chondrocyte differentiation (Garciadiego-Cazares et al., 2015). Treatment of differentiating mesenchymal stem cells with TGF-β superfamily member GDF-5 induced α5 integrin expression and prevented chondrocyte hypertrophy (Garciadiego-Cazares et al., 2015). It was demonstrated that in articular cartilage of rats with surgically induced OA α5 integrin expression was reduced and associated with chondrocyte hypertrophy. It was concluded that α5 integrin expression is protective against hypertrophic changes.

In conclusion, these studies further establish that different TGF-β superfamily members are involved in regulating the chondrocyte phenotype *via* tuning of different downstream factors involved in gene transcription, which ultimately leads to either chondroprotective or pro-hypertrophic responses.

### MAP Kinases

**Figure 4:**
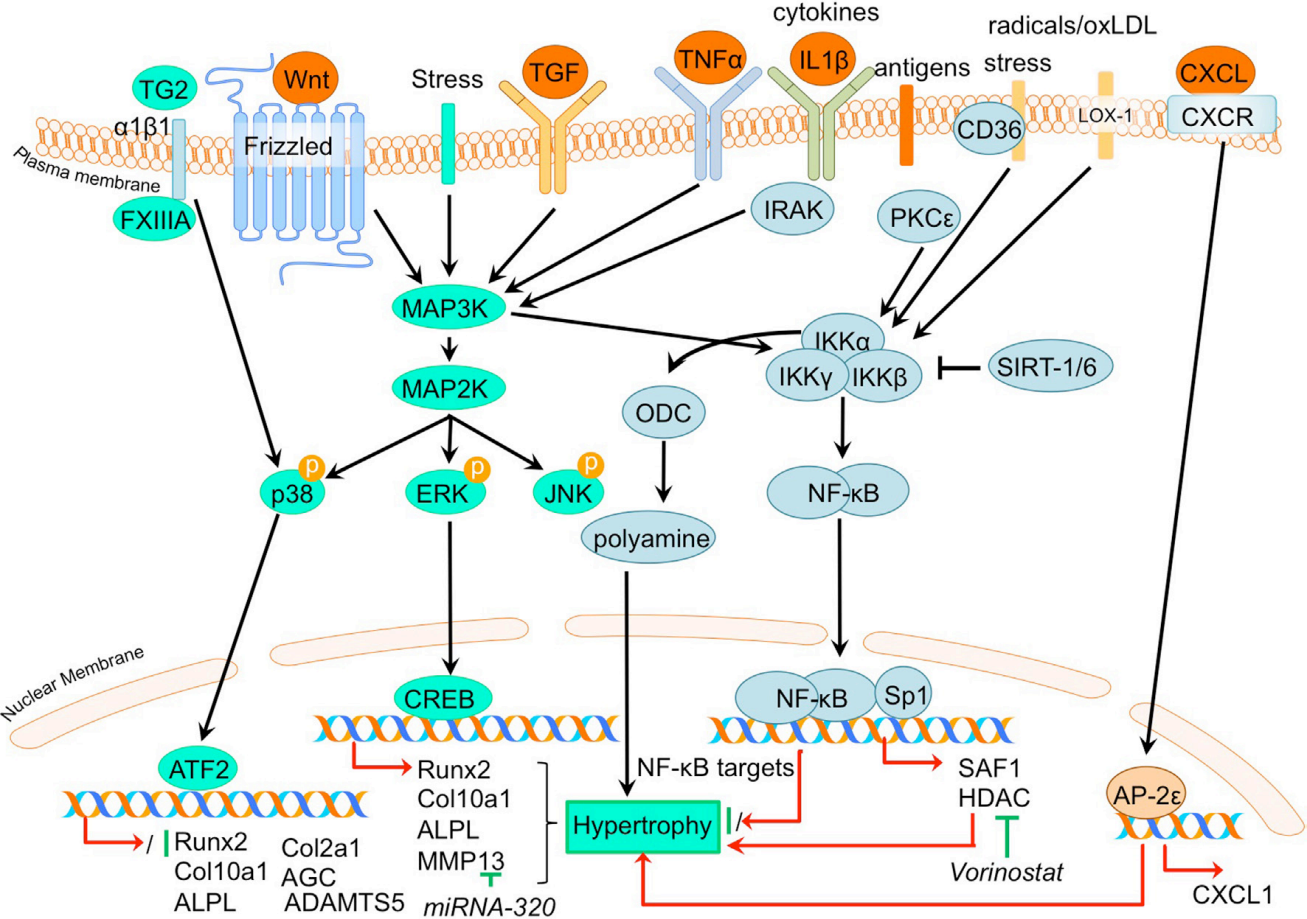
Overview of the canonical MAP kinase (MAPK) and inflammatory signaling pathways and newly acquired insights into their relation to the development of the osteoarthritic (OA) chondrocyte hypertrophic phenotype. The canonical pathway was updated with recent insights in the role of MAPK and inflammatory signaling in the hypertrophic switch occuring during OA development. In green pro-chondrogenic relations are shown, while pro-hypertrophic relations are depicted in red.

The MAP kinase (MAPK) pathway consists of a sequence of intracellular signaling proteins which transduce a signal from various cell receptors to the nucleus (Schaeffer and Weber, 1999). Activity of MAPKs is regulated in response to environmental stress and to cytokines and growth factors, such as members of the Wnt family (Bikkavilli and Malbon, 2009) or the TGF-β superfamily (Derynck and Zhang, 2003). After activation, a cascade of phosphorylating events takes place intracellularly, finally resulting in phosphorylation of the MAPKs themselves. The three major MAPK pathways, include p38, c-Jun N-terminal (JNK) kinase, and extra-cellular-regulated kinases (ERK) (Johnson and Lapadat, 2002). The currently held doctrine considers MAPKs as signaling mediators involved in the endochondral ossification process (Stanton et al., 2003), but they are also able to regulate the activity of multiple mediators of cartilage destruction (Loeser et al., 2008). Studies suggest that the ERK pathway is involved in destructive OA responses (Prasadam et al., 2013), while the p38 pathway is OA protective (Li et al., 2010; Prasadam et al., 2010). An overview of the newly acquired insights into this pathway and its involvement in development of OA chondrocyte hypertrophy is provided in Table 1d and Figure 4.

It was demonstrated that phosphorylation of ERK1/2 was increased in OA tibial cartilage together with an increased hypertrophic phenotype, characterized by increased COL10A1 and RUNX2 expression levels in a surgically induced rat OA model (Prasadam et al., 2013). On the other hand, expression levels of phosphorylated p38 were decreased in OA compared to non-OA cartilage (Prasadam et al., 2013). This study also revealed that pharmacological ERK inhibition together with hyaluronic acid treatment resulted in a synergistic chondroprotective effect compared to hyaluronic acid treatment only. This was characterized by significantly reduced Mankin scores, accompanied by reduced COL10A1 expression levels (Prasadam et al., 2013). Higher pERK levels, along with increased expression levels of hypertrophic markers, such as *ALPL* and *RUNX2* were also found in OA articular chondrocytes compared to healthy articular chondrocytes (Prasadam et al., 2010). Furthermore, VEGF-induced hypertrophy of chondrocytes was accompanied by pERK activation (Zhang et al., 2016a,b).

In contrast to pERK, p38 has been negatively associated with OA. P38 expression levels were higher in wild-type murine articular cartilage compared to *Smad3* knockout mice displaying cartilage damage (Li et al., 2010). P38 levels were also lower in human OA articular chondrocytes compared to healthy human articular chondrocytes (Prasadam et al., 2010).

The pro-hypertrophic effects of pERK and contra-hypertrophic effects of p38 were further substantiated in a co-culture system using subchondral osteoblasts (Prasadam et al., 2010). Co-culturing human articular chondrocytes with subchondral osteoblasts induced hypertrophic changes in the articular chondrocytes, evidenced by increased *RUNX2, ALPL*, and *COL10A1* expression and decreased *COL2A1* and *AGC* expression. Hypertrophic induction in these articular chondrocytes was accompanied by increased pERK phosphorylation and phosphorylation of p38 was decreased. Inhibition of pERK in cultures was able to reduce the hypertrophic induction of articular chondrocytes, in contrast to inhibition of p38 that resulted in hypertrophic induction (Prasadam et al., 2010). Furthermore, confirming a role for pERK in hypertrophic processes in OA chondrocytes, ERK1/2 inhibition abolished FGF23-induced *MMP13* expression (Bianchi et al., 2016).

Although p38 is generally considered to act in a chondroprotective manner, it has been evidenced that under certain conditions the p38 activation may result in hypertrophic differentiation in cultured chondrocytes (Merz et al., 2003; Wang and Beier, 2005). In a study by Johnson et al. (2008) it was found that the transglutaminases (TG) TG2 and factor XIIIA (FXIIIA) were increased in hypertrophic chondrocytes from the growth plate and in OA articular chondrocytes (Johnson et al., 2008). Externalization of TG2 is mediated by FXIIIA *via* its interaction with α1β1 integrins and results in activation of the p38 MAPK signaling pathway, which ultimately led to increased COL10A1 expression in this study (Johnson et al., 2008). Additionally, TG2 expression was found to be associated with enhanced articular chondrocyte hypertrophy in a Hartley Guinea Pig Model of OA as determined by increased MMP13 and ADAMTS5 and an enhanced *Col10a1:Col2a1* ratio (Huebner et al., 2009).

Taken together, specific MAP kinases have been demonstrated to regulate both hypertrophic and chondrogenic responses in the chondrocyte and inhibition of specific MAP kinases could potentially be a strategy to block OA progression *via* modulating the hypertrophic chondrocyte phenotype.

### Inflammatory Signaling

Not surprisingly, regarding the inflammatory nature of OA, the involvement of the NF-κB (nuclear factor kappa-light-chain-enhancer of activated B cells) pathway has been described as involved in the regulation of hypertrophic differentiation in OA (Marcu et al., 2010). Many stimuli (such as stress, cytokines, free radicals, heavy metals, ultraviolet irradiation, oxidized LDL, and bacterial or viral antigens), activate NF-κB, mostly through IκB kinase-dependent (IKK-dependent) phosphorylation and subsequent degradation of NF-κB inhibitory IκB proteins. The liberated NF-κB dimer enters the nucleus, where it regulates transcription of diverse target genes (Marcu et al., 2010). An overview of newly acquired insights into this pathway and its involvement in development of OA chondrocyte hypertrophy is provided in Table 1e and Figure 4.

Providing evidence for a role of NF-κB signaling in chondrocyte hypertrophy, it was demonstrated that IKKα and IKKβ knockdown in OA chondrocyte micromass cultures results in an increased GAG content, COL2A1 expression, and reduced calcium deposits. IKKα and IKKβ also appeared to have differential activities, since IKKα knockdown, but not IKKβ knockdown, resulted in smaller OA hypertrophic chondrocytes (Olivotto et al., 2008). Furthermore, only IKKα knockdown resulted in reduced RUNX2 levels, while IKKβ knockdown resulted in increased SOX9 levels. The pro-hypertrophic role of IKKα was later confirmed by the same group (Olivotto et al., 2013), as well as another group (Facchini et al., 2012). Facchini et al. revealed that expression of IKKα is related to chondrocyte hypertrophy and knockdown of IKKα results in reduced synthesis and activity of ornithine decarboxylase (ODC). ODC mediates the increase of polyamine levels that in turn increases *RUNX2* expression, as well as RUNX2 nuclear translocation, leading to hypertrophic chondrocytes (Facchini et al., 2012). Additionally, a study by Meng et al. also revealed a role for NF-kB signaling in chondrocyte hypertrophy (Meng et al., 2016). In this study it was found that microRNA- (MiRNA)320 negatively regulates *MMP13* expression by binding to the 3’UTR (3’ untranslated region) of the *MMP13* mRNA. In turn, miRNA320 expression is reduced by IL-1β-induced NF-κB and MAPK signaling activity (Meng et al., 2016). This study thus revealed a link between a hypertrophy-dependent decrease of miRNA-320 and an increase of *MMP13 via* the NF-κB and MAPK signaling pathway. Oxidized low-density lipoprotein (ox-LDL) binding to lectin-like ox-LDL receptor-1 (LOX-1) in cultured bovine articular chondrocytes increased production of intracellular reactive oxygen species (ROS), resulting in the activation of the inflammatory signaling pathway *via* NF-κB (Nishimura et al., 2004). Hashimoto et al. demonstrated a role for LOX-1 in chondrocyte hypertrophy, as COL10A1 expression in a DMM mice OA model was induced by expression of the LOX-1. This resulted in decreased articular cartilage GAG content (Hashimoto et al., 2016). *LOX-1*^−/−^ mice on the other hand, revealed a reduced OA score and a reduction in osteophyte formation. LOX-1 co-localized with RUNX2 and COL10A1 expression in articular chondrocytes as well as in osteophyte forming-cells, indicating a role in the pathogenesis of DMM-induced OA through endochondral ossification (Hashimoto et al., 2016).

In a study by Zhong et al. it was found that in IL-1β-treated OA chondrocytes, expression of the NF-κB subunit p65 was reduced in response to the HDAC-inhibitor vorinostat. Besides this inflammatory pathway, HDAC inhibition by vorinostat also resulted in decreased p38 and ERK1/2 activation in IL-1β-exposed human OA chondrocytes (Zhong et al., 2013). During OA progression in human articular chondrocytes, PKCε (protein kinase C epsilon) levels were reduced, which lead to an upregulation of *HDAC2* and reduction of *HDAC4* (Queirolo et al., 2016). This resulted in an increase of the *HDAC2:HDAC4* ratio, which then induced RUNX2 and ultimately resulted in increased expression of MMP13, ADAMTS4, and ADAMTS5 (Queirolo et al., 2016). Interestingly, the decreased expression of HDAC4, related to the loss of PKCε in OA chondrocytes (Queirolo et al., 2016), was confirmed in another study by Lu et al. (2014a). However, in contradiction to the previous study, Lu et al. found that silencing of *HDAC4* resulted in a decrease of chondrocyte hypertrophic marker expression, while additionally HDAC4 was decreased with increasing severity of OA, suggesting a role for HDAC4 in the onset of OA.

Besides NF-κB, ELR + CXC chemokines, which are characterized by their glutamic acid-leucine-arginine (ELR+) motif, provide new links connecting OA to hypertrophic changes (Wenke et al., 2011). AP-2ε is a transcription factor which acts in hypertrophic cartilage differentiation and its expression is increased in OA chondrocytes compared to primary chondrocytes (Wenke et al., 2009). This study described that *CXCL1* expression accompanied the increase in *AP-2*ε expression levels during hypertrophic differentiation of MSCs (Wenke et al., 2011). Expression of *CXCL1* appeared to be under transcriptional control of AP-2ε, since AP-2ε was demonstrated to bind and transactivate the *CXCL1* promotor, indicating that the observed increase in AP-2ε in OA chondrocytes may lead to hypertrophic changes *via* regulating CXCL1 activity (Wenke et al., 2011). Another chemokine that was revealed to be involved in OA pathogenesis is CXCL6 (Sherwood et al., 2015). It was shown that the expression of this chemokine is reduced in human OA cartilage (Sherwood et al., 2015) and also in a mouse DMM OA model (Sherwood et al., 2015). Knockout of the CXCL6 receptor, *CXCR2*, resulted in more severe OA development after DMM surgery, as evidenced by higher OARSI scores (Sherwood et al., 2015). This was also accompanied by a more hypertrophic phenotype verified by increased COL10A1 protein expression. These data suggest a chondroprotective action of the CXCL6-CXCR2 axis.

In addition to chemokines, different inflammatory cytokines have been implicated in OA pathophysiology, one of them being TNFα (Lai et al., 2014). It was recently confirmed that TNFα induces *Adamts7* expression in murine cartilage, and ADAMTS7 was able to induce the expression of *Tnf*α, creating a positive feedback loop (Lai et al., 2014). TNFα-mediated transactivation of an *Adamts7* promotor reporter construct was dose-dependently inhibited by Bay 11-7082, a NF-κB-specific inhibitor, indicating NF-κB-mediated activation of *Adamts7* after TNFα stimulation. Interestingly, mice overexpressing ADAMTS7 displayed OA-like phenotypes characterized by reduced cartilage GAG content, osteophyte formation, thinner cartilage, and an upregulation of hypertrophic marker expression such as Col10a1 and MMP13. Furthermore, ADAMTS7 overexpression resulted in an acceleration of OA development in a mouse DMM experiment which was also accompanied by increased expression of chondrocyte hypertrophic markers *Col10a1* and *Mmp13* (Lai et al., 2014). The ADAMTS7 overexpressing mice also displayed several skeletal developmental abnormalities, including a reduced hypertrophic zone and reduced COL10A1 levels in the growth plate and lower bone mineral density.

The inflammatory S100A11, a ligand for the receptor for advanced glycation end products (RAGE), is associated with chondrocyte hypertrophy (Cecil et al., 2009). However, the deletion of RAGE was not chondroprotective in an instability induced knee OA mouse model (Cecil et al., 2009). Cecil et al. (2009) demonstrated that the alternative patterning receptor CD36, a marker for growth plate chondrocyte hypertrophy, promotes OA *via* mediation of inflammatory and differentiation responses. Indeed, CD36 co-localized with COL10A1 expression in all zones of knee articular cartilage, as well as the aggrecan NITEGE aggrecanase neoepitope in the articular cartilage superficial zone. Surprisingly, overexpression of CD36 in CH-8 cells led to an inhibition of chondrocyte hypertrophic markers (Cecil et al., 2009), while S100A11 gained the capacity to induce proteoglycan synthesis in CH-8 chondrocytes (Cecil et al., 2009). These results indicate that CD36 is a hypertrophic chondrocyte-expressed patterning receptor that induces cartilage repair when exposed to inflammatory stimuli (Cecil et al., 2009). This demonstrates that besides pro-hypertrophic processes, inflammatory responses in articular chondrocytes can also result in a chondroprotective effect.

The semicarbazide-sensitive amine oxidase (SSAO) found in the hypertrophic chondrocytes of the growth plate is known to be involved in leukocyte extravasation from the blood to the inflammation site. Additionally, it may be associated with the differentiation of chondrocytes toward a hypertrophic phenotype (Filip et al., 2016). In line with this, inhibition of SSAO reduced *Mmp13, Alpl*, and *Opn* expression, potentially by modulation of glucose transport in rat hypertrophic articular chondrocytes (Filip et al., 2016). In humans, similar expression patterns were seen for SSAO in healthy and OA articular cartilage, supporting the idea that SSAO plays a role in the process of hypertrophic differentiation of the articular chondrocyte (Filip et al., 2016). OA is a disease associated with aging, which could be a result of tissue accumulation of p16^INK4a^ positive cells. p16^INK4a^ is suggested to support chronic inflammation, as p16^INK4a^ positive cells exhibit a specific secretome called SASP (senescence-associated secretory phenotype) including pro-inflammatory cytokines. OA chondrocytes are characterized by an accumulation of p16^INK4a^ as a result of reduced *miRNA24* levels, which acts as a negative regulator for p16^INK4a^ (Philipot et al., 2014). p16^INK4a^ is also upregulated during chondrogenesis, indicating a recapitulation of a developmental process in OA.

Since inflammation and angiogenesis are closely correlated in the pathogenesis of OA, Ray et al. hypothesized the presence of common regulators controlling both processes simultaneously (Ray and Ray, 2008). In their study they found that overexpression of the inflammation responsive transcription factor SAF-1 in transgenic mice leads to the development of severe cartilage degradation and OA. SAF-1-overexpressing mice also showed neo-vasculature in the perichondrium and synovium, suggesting a link between angiogenesis and inflammation. Indeed, the *VEGF* promotor contains two tandem binding sites for SAF-1 (Ray and Ray, 2008), providing evidence for the link between inflammation and OA development and pathogenesis. Furthermore, SAF-1 has binding sites in the *MMP1* and *MMP9* promotor sequences (Ray and Ray, 2008), controlling their expression. In human OA articular chondrocytes, SAF-1 expression is increased and together with NF-κB and SP1 acts synergistically to induce MMP activation in OA chondrocytes.

Another factor involved in inflammatory signaling and OA disease progression is SIRT-1. SIRT-1 inhibits NF-κB by deacetylating the p65 NF-κB subunit, priming p65 for proteasome degradation, thus modulating the inflammatory signaling pathway (Liu-Bryan, 2015). The association of SIRT-1 with chondrocyte hypertrophy was demonstrated after inhibition of SIRT-1 by RNAi, which induced *COL10A1* expression. Since OA chondrocytes display decreased SIRT-1 expression compared to healthy controls, this may provide a route for chondrocytes to acquire an endochondral cellular phenotype (Fujita et al., 2011). Besides SIRT-1, SIRT-6 also modulates inflammation. *SIRT-6* haplo-insufficiency enhanced OA progression in high fat diet-induced obese mice by stimulating the inflammatory response as shown by increased expression of *Tnf*α and *IL-6* in the HFP (Ailixiding et al., 2015). Additionally, *SIRT-6* haplo-insufficient mice on a high fat diet revealed an increase in osteophytes and synovial tissue with increased infiltration of inflammatory cells, as well as increased MMP13 expression in the cartilage. These results indicate a higher inflammation status of the HFP and synovium in obese mice with *SIRT-6* haplo-insufficiency accompanied with increased osteophyte formation, leading to OA.

To conclude cytokines and chemokines and their downstream intracellular pathways have been revealed to lead to hypertrophic as well as anti-hypertrophic changes in the chondrocyte. While inflammation-induced hypertrophic changes have been established as associated with processes involved in cartilage matrix degradation, inflammation-induced anti-hypertrophic changes have been described to lead to chondroprotective responses.

## Hypoxic and Angiogenic Factors

**Figure 5:**
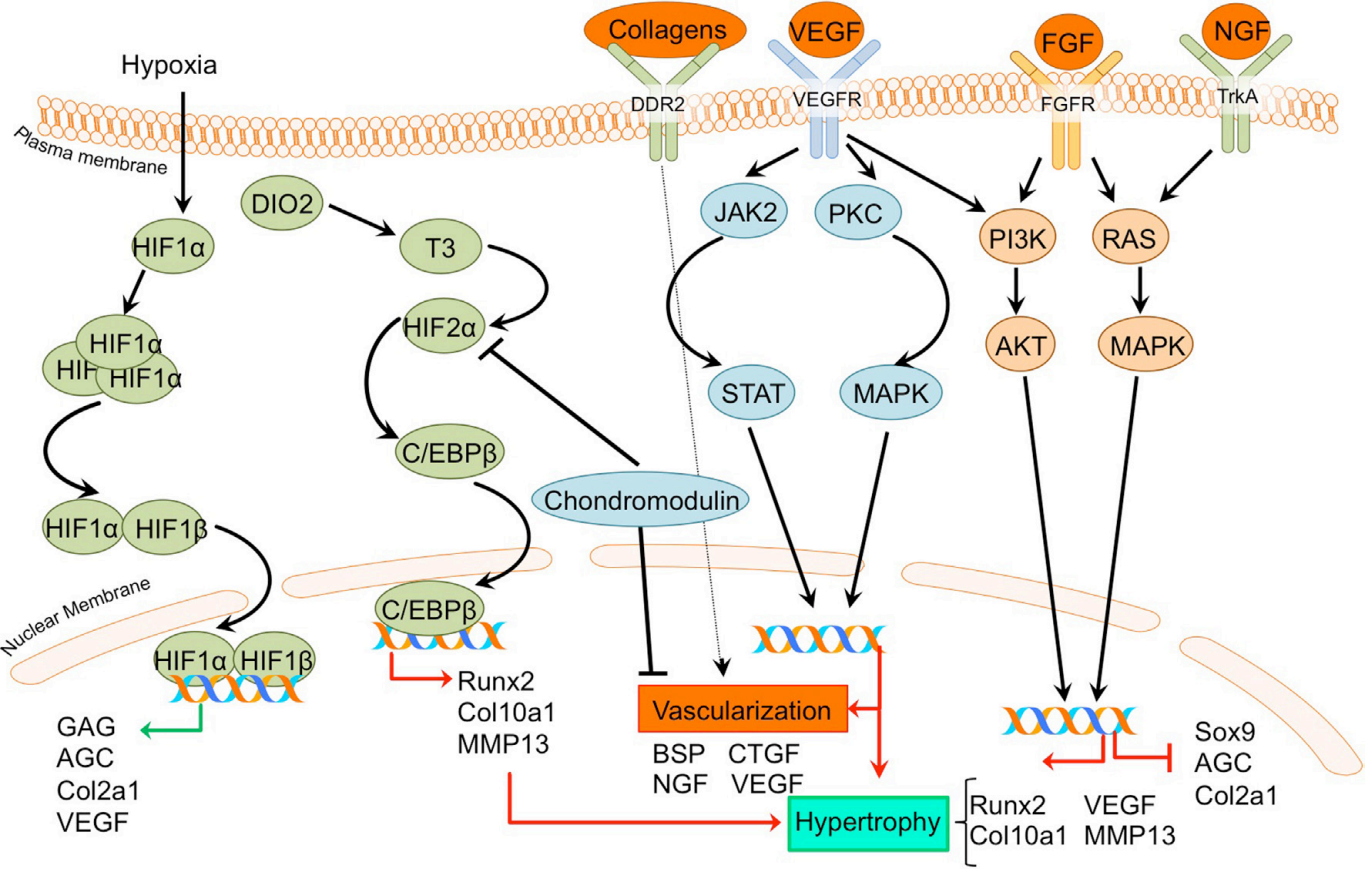
Overview of the processes of hypoxia and angiogenesis and the canonical fibroblast growth factor (FGF) signaling pathway and newly acquired insights into their relation to the development of the osteoarthritic (OA) chondrocyte hypertrophic phenotype. The processes and canonical FGF signaling pathway was updated with recent insights in the role of the processes of hypoxia and angiogenesis and the FGF signaling pathway in the hypertrohic switch occuring during OA development. In green pro-chondrogenic relations are shown, while pro-hypertrophic relations are depicted in red.

The proliferative zone of the growth plate is an avascular hypoxic mesenchymal tissue (Maes et al., 2012). Intriguingly, chondrocytes are competent at surviving and differentiating in this challenging environment. It has been suggested that this survival and capability of differentiation is, at least in part, by virtue of the actions of hypoxia-inducible factors (HIFs), such as HIF1-α (Maes et al., 2012). Furthermore, the process of endochondral ossification in the growth plate is driven by vascularization and hypertrophic chondrocytes in the growth plate that secrete angiogenic stimuli which actively support vascularization (Maes et al., 2012). This hypertrophy associated angiogenic switch is a major driver of active growth plate cartilage remodeling toward bony tissue (Maes et al., 2012). The analogy between endochondral ossification processes and molecular processes observed in OA also led to the theory that OA pathophysiology may involve hypoxic/angiogenic mediators, such as HIFs and VEGF. An overview of the newly acquired insights into this pathway and its involvement in development of OA chondrocyte hypertrophy is provided in Table 1f and Figure 5.

A direct link between HIF-2α and OA development became evident by a study reported by Saito et al (Saito et al., 2010). It revealed that HIF-2α is localized predominantly in the hypertrophic zone of mouse growth plates, and *Hif-2*α^+/−^ mice display reduced proliferative and hypertrophic zone lengths, together with impaired bone length, indicating impaired endochondral ossification during limb development. Confirming its role in OA pathophysiology, HIF-2α expression was increased in articular chondrocytes of a surgically induced OA mouse model, while *Hif-2*α^+/−^ caused significant resistance to cartilage degradation, osteophyte formation, and subchondral bone sclerosis in this model (Saito et al., 2010). Furthermore, in the same study HIF-2α expression was revealed to be higher in more severely OA-affected human cartilage samples than in mild OA affected regions. The crucial role of HIF-2α in OA pathophysiology was confirmed in another study, which described that *Hif*-2α^+/−^ mice display less cartilage degradation in a surgically induced mouse OA model (Hirata et al., 2012). Furthermore, it was evident that in chondrocytes, HIF-2α is a potent inducer of C/EBPβ (Hirata et al., 2012). Confirming a role for C/EBPβ in OA pathophysiology, *C/Ebp*β^+/−^ mice developed less severe OA cartilage damage in a surgically induced mouse OA model (Hirata et al., 2012). Furthermore, C/EBPβ together with RUNX2 is higher expressed in human OA cartilage with higher Mankin scores compared to human OA cartilage with lower Mankin scores, and can activate *MMP13* expression in chondrocytes (Hirata et al., 2012).

*Dio2* is upregulated in OA cartilage (Nagase et al., 2013) and is responsible for active thyroid hormone (T3) production. T3 in turn induces terminal chondrocyte differentiation with increased HIF-2α expression, as well as *COL10A1, ALPL, osteocalcin, RUNX2, MMP13*, and *ADAMTS5* expression (Nagase et al., 2013; Bomer et al., 2015). The upregulation of *HIF-2*α expression after T3 treatment suggests a link between DIO2 levels and OA development *via* HIF-2α signaling and activating mutations in the *DIO2* allele result in a predisposition for OA development in human patients (Bomer et al., 2015). Additionally, it was demonstrated in an experimental *Dio2* transgenic rat OA model that overexpression of DIO2 results in increased OA-associated cartilage degradation with higher COL10A1 expression levels (Nagase et al., 2013). Confirming parallels between chondrocyte signaling events that take place in the growth plate and those of hypertrophic chondrocytes in OA-affected articular cartilage, DIO2 activity was also increased during chondrogenesis of BMSCs (Bomer et al., 2015).

Despite the general recognition that HIF expression in cartilage is associated with OA disease progression, a study by Markway et al. could not detect a differential expression of HIF-1α and HIF-2α in OA versus healthy chondrocytes under hypoxic conditions (Markway et al., 2013). In this study, hypoxia-induced HIF expression resulted in increased COL2A1, ACAN, and GAG levels (Markway et al., 2013). While HIF-2α is normally described as associated with hypertrophy, HIF-3α is not. HIF-3α expression is higher in the resting and proliferative zones of the growth plate compared to the hypertrophic zone (Markway et al., 2015). Furthermore, chondrogenically differentiated BMSCs showed *MMP13* and *COL10A1* induction, while *HIF-3*α expression was low.

In addition to HIF-2α, VEGF was demonstrated to be involved in OA development. In a surgically induced rat OA model, inhibition of *Vegf* with a shRNA resulted in less cartilage degradation compared to rats without *Vegf* inhibition at 5–9 weeks after OA induction. This chondroprotective effect was accompanied by reduced *Col10a1* levels (Zhang et al., 2016a).

Another protein essential in vascularization during endochondral ossification is chondromodulin. Proliferative chondrocytes in the growth plate are resistant to vascular invasion because of the presence of angiogenic inhibitors, such as chondromodulin (ChM-1) (Hiraki and Shukunami, 2005). Chondromodulin overexpression protected against OA development in a surgically induced rat OA model, evidenced by lower Mankin scores, less COL10A1 expression and higher COL2A1 and AGC levels (Zhang et al., 2016a,b). Likewise, *Chm-1* levels in OA cartilage were lower in a surgically induced rat OA model compared to cartilage from control joints (Zhang et al., 2016b). Furthermore, Chm-1 expression was decreased in more severely damaged human OA cartilage in comparison to mild OA cartilage (Zhang et al., 2016b). Chondromodulin also protected against TNFα-induced chondrocyte hypertrophy (Zhang et al., 2016b). Additional experiments in the C28/I2 chondrocyte cell line revealed that chondromodulin delays HIF-2α nuclear translocation, indicating that the anti-hypertrophic and chondroprotective effects of chondromodulin are caused by the repression of pro-hypertrophic HIF-2α activity (Zhang et al., 2016b).

Vascularization is essential for the endochondral ossification process and it has also been associated with OA. Indeed, OA cartilage is invaded by blood vessels into the non-calcified articular cartilage, likely due to an increased production of pro-angiogenic factors (Mapp and Walsh, 2012; Wang et al., 2012). This has been attributed to the increase in subchondral bone porosity (Botter et al., 2011), which has been hypothesized to result in disruption of the osteochondral junction. A disruption of the osteochondral junction with a subsequent invasion of blood vessels may lead to further structural damage, leading to progression of OA.

A role for chondrocyte hypertrophic processes in OA progression was further described by a study in which conditioned medium of hypertrophic OA chondrocytes induced wound healing in human umbilical vascular endothelial cells and increased endothelial cell adhesion and migration (Pesesse et al., 2013). Furthermore, the gene expression of pro-angiogenic factors, such as *BSP* and *NGF* was increased (Pesesse et al., 2013). Osteochondral angiogenesis was also assessed in a study by Wang et al. in which OA was evoked by disordered occlusion in rat mandibular joints (Wang et al., 2012). Experimental groups demonstrated OA-like changes, with a loss of cartilage surface integrity and osteophyte formation. Additionally, hypertrophic chondrocytes adjacent to the osteochondral interface showed increased expression of VEGF, CTGF, and MMP9 at 20 or 24 weeks post OA-induction surgery. Thus, hypertrophic chondrocytes may exacerbate the disruption of the osteochondral junction by stimulating angiogenesis, which can lead to progression of OA. Another study described the association between pro-angiogenic factors, hypertrophy, and OA severity. Discoidin domain receptor 2 (DDR2) was higher expressed in more severely damaged human OA cartilage compared to cartilage with lower Mankin scores (Zhang et al., 2014a). DDR2 is a receptor tyrosine kinase that can be activated by various collagens. Since DDR2 activity can induce COL10A1 expression in chicken chondrocytes (Zhang et al., 2014b), and given the fact that DDR2 has been associated with a pro-angiogenic function (Zhang et al., 2014a), this supports a link between chondrocyte hypertrophic pro-angiogenic factors and OA progression.

In conclusion, these results reveal the importance of angiogenesis in enabling OA disease progression. The differential activation of hypoxic and angiogenic pathways observed in OA appear to be key factors in OA development.

### FGF Signaling

Fibroblast growth factors comprise a group of morphogens involved in wound healing, angiogenesis, and are involved in processes such as proliferation and differentiation in different cell types (Turner and Grose, 2010). They also have been described as being involved in endochondral ossification, since FGF23 and FGF receptor 1 are produced by growth plate hypertrophic chondrocytes (Raimann et al., 2013). Given that OA chondrocytes also produce FGF family members (Orfanidou et al., 2009), studies have investigated the involvement of FGF signaling in the chondrocyte phenotypic alterations observed in OA cartilage. An overview of newly acquired insights into this pathway and its involvement in development of OA chondrocyte hypertrophy is provided in Table 1g and Figure 5.

It has been demonstrated that the expression of FGFR1, FGF23, and its co-receptor KLOTHO is higher in OA chondrocytes compared to non-OA chondrocytes. Expression of these factors was also increased in cartilage samples with more severe macroscopic OA compared to less severe macroscopic OA within the same patient (Bianchi et al., 2016). Exogenous addition of FGF23 to human OA primary chondrocytes resulted in hypertrophic changes, as evidenced by *COL10A1* and *VEGFA* induction *via* FGFR1 (Bianchi et al., 2016). Another study confirmed the increase in FGF23 expression levels in OA compared to non-OA chondrocytes (Orfanidou et al., 2009). This study confirmed the hypertrophic phenotype in OA chondrocytes with higher RUNX2 and lower SOX9 levels compared to non-OA chondrocytes, and also revealed that exogenous addition of FGF23 to non-OA chondrocytes induced a hypertrophic phenotype characterized by increased RUNX2 expression levels (Orfanidou et al., 2009). Involvement of FGF signaling as an OA-inducing pathway was demonstrated in a study in which G141, a pharmacological FGFR1 inhibitor was used. In a surgically induced mouse OA model, G141 delayed the progression of cartilage degradation, accompanied by a decrease of MMP13 and COL10A1 chondrocyte hypertrophic marker expression, suggesting that OA articular cartilage damage could be reduced *via* inhibition of hypertrophic processes (Xu et al., 2016).

On the other hand, FGFR3 seems to be crucial for cartilage homeostasis and inhibition of a hypertrophic phenotype, since FGFR3 knockout mice displayed OA-like defects in the temporomandibular joint together with an increased expression of hypertrophic markers COL10A1 and MMP13 (Zhou et al., 2016). Further support indicative of an association between chondrocyte hypertrophy and FGFR3 comes from a study in which it was revealed that FGFR3 levels were reduced in *mtorc1* knockout mice which display cartilage degradation and chondrocyte hypertrophy (Zhang et al., 2017).

Taken together, recent studies have demonstrated that the FGF signaling pathway is active in OA and also provided new insights in the contribution of this signaling pathway to OA disease initiation or progression.

### Notch Signaling

The Notch signaling pathway consists of five identified ligands (Jagged 1, Jagged 2, Dll1, Dll3, and Dll4) that can interact with four receptors (Notch 1–4) (Bray, 2016). Upon binding of Notch ligands, the Notch receptor cleavage site becomes accessible for cleavage by ADAM10. The residual transmembrane Notch fragment in turn is cleaved *via* proteolysis by the γ-secretase complex, releasing NICD. This intracellular domain of the receptor translocates to the nucleus, where it binds to the transcription factor recombination signal binding protein for immunoglobulin kappa J (RBPjκ), to participate in gene transcription of Notch target genes, such as *Hes1*. These target genes are involved in cellular processes, such as cell proliferation and differentiation (Nye and Kopan, 1995). Notch signaling is involved in endochondral ossification (Hosaka et al., 2013), and, therefore, studies have investigated its involvement in OA development as well. An overview of newly acquired insights into this pathway and its involvement in development of OA chondrocyte hypertrophy is provided in Table 1h and Figure 3.

Providing evidence for a role of Notch signaling in endochondral processes and OA development, it has been reported that RBPjκ is involved in endochondral ossification. Knockout of *Rbpj*κ resulted in reduced cartilage damage in a surgically induced OA model, together with reduced expression of MMP13, VEGFA, and HES1 in articular chondrocytes (Hosaka et al., 2013). Furthermore, in this study it was revealed that during OA development, the expression of Notch ligand Jagged1 is increased in OA cartilage, indicating that this ligand may regulate Notch signaling during OA progression (Hosaka et al., 2013). Confirming again a role for Notch signaling in OA development, it was demonstrated that the pharmacological Notch signaling inhibitor *N*-[*N*-(3,5-diflurophenylacetate)-l-alanyl]-(*S*)-phenylglycine t-butyl ester (DAPT), resulted in the same chondroprotective effect in a surgically induced mouse OA model (Hosaka et al., 2013), as observed with the *Rbpj*κ knockout mouse.

In concert with a role in chondrocyte hypertrophy, the intracellular domains of Notch ligands, NOTCH1 and NOTCH2 was shown to be located at the chondrocyte plasma membrane in resting and proliferative zone chondrocytes, while these intracellular domains were translocated (and thus potentially active) to the nucleus in hypertrophic zone chondrocytes (Hosaka et al., 2013). Similar as in the growth plate’s hypertrophic zone, the intracellular domains of NOTCH1 and NOTCH2 were also located to the chondrocyte’s nucleus in surgically induced mouse OA cartilage (Hosaka et al., 2013). An increase in Notch signaling activity was also observed in a further study. In this study, an increase in mRNA levels of Notch ligand *Jagged 1* and its receptor *Notch 1* in OA areas of human articular cartilage, together with induced mRNA levels of the Notch target gene *HES1* was reported (Lin et al., 2016). Interestingly, inhibition of NOTCH1 resulted in an increased, HES1 dependent, hedgehog signaling activity, leading to an exacerbation of OA (Lin et al., 2016). These results are in contrast with the general theory that inhibition of Notch attenuates OA development. The increase in hypertrophy may be a result of a crosstalk between the Notch and hedgehog signaling pathway, previously described in the Ihh/PTHrP signaling paragraph.

In conclusion, these results provide evidence that the Notch signaling pathway is positively associated with hypertrophic changes in the chondrocyte. Inhibition of the Notch signaling pathway may be used as a therapeutic tool to block OA disease progression, based on reducing hypertrophic changes in the chondrocyte.

### Mineralization

**Figure 6:**
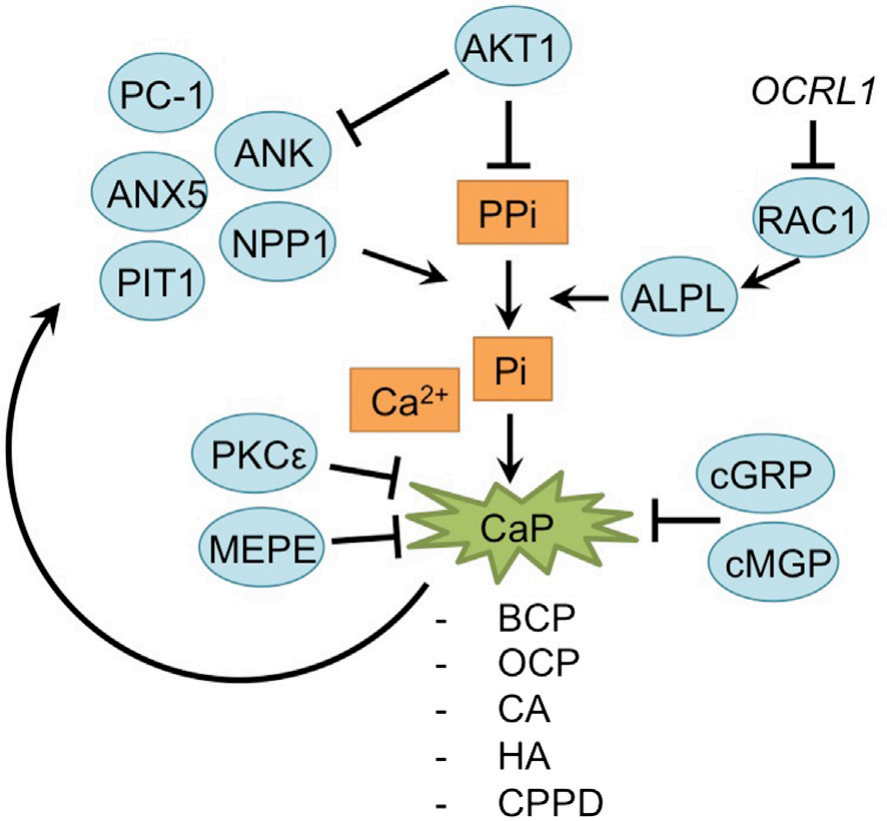
Overview of the mineralization process and newly acquired insights into its relation to the development of the osteoarthritic (OA) chondrocyte hypertrophic phenotype. The mineralization process was updated with recent insights in the role of mineralization in the hypertrophic switch occuring during OA development.

Mineralization is also an important consequence of chondrocyte hypertrophy in OA (Fuerst et al., 2009; Fukai et al., 2010; Wallin et al., 2010; Nguyen et al., 2013; Zhu et al., 2015; Cavaco et al., 2016; Nasi et al., 2016; Queirolo et al., 2016). The mineralization process is analogous to the last phase of endochondral ossification as it is observed in the growth plate (Kronenberg, 2003). Here, hypertrophic chondrocytes secrete matrix vesicles containing high concentrations of phosphatases, such as alkaline phosphatase (ALPL) and PHOSPHO1 (Anderson, 2003; Houston et al., 2004; Stewart et al., 2006; McKee et al., 2013). During matrix vesicle biogenesis, vesicles are formed by polarized budding and pinching-off of vesicles from the plasma membranes. Within these matrix vesicles, the first mineral crystals are formed by phosphatases hydrolyzing inorganic pyrophosphate (PPi) to create inorganic phosphate (Pi). Pi ions in turn crystallize with calcium, resulting in crystals which are released through the vesicles membranes. When these pre-formed hydroxyapatite crystals come in contact with the extracellular fluid containing Ca^2+^ and $PO_{4}^{3-}$ ions a process of continuous crystal formation takes place in the matrix (Anderson, 2003; Orimo, 2010). This mineralized matrix is then vascularized, further supporting mineralization and enabling the infiltration of osteoblasts and osteoclasts. Osteoblasts secrete osteoid, which forms the bone trabecula, while osteoclasts, formed from macrophages, breakdown spongy bone to form the medullary (bone marrow) cavity (Kronenberg, 2003). An overview of newly acquired insights into this process and its involvement in development of OA chondrocyte hypertrophy is provided in Table 1I and Figure 6.

During endochondral ossification, AKT1 induces mineralization without affecting the hypertrophic and proliferative zone in the growth plate (Fukai et al., 2010). Surgically DMM OA-induced *Akt1*^−/−^ mice specifically revealed a reduction of calcified osteophyte formation, while cartilage degradation was unaltered, supporting results observed in the growth plate. This may be the result of an increase of inorganic pyrophosphate (PPi) due to *Akt1* inhibition, which antagonizes the ability of inorganic phosphate (Pi) ions to crystallize with calcium. Indeed, levels of PPi-regulators *Ank* and *Npp1* were increased after *Akt1* inhibition (Fukai et al., 2010).

A further relationship was found between RAC1 and hypertrophy (Zhu et al., 2015). Lenti-viral expression of the RAC1 inhibitor OCRL1 resulted in protection against cartilage degradation in a mouse OA model. Additionally, OCRL1 resulted in decreased ALPL activity in human primary chondrocytes. Pre-treatment of chondrocytes with IL-1β, resulted in an upregulation of ADAMTS5, Col10a1, RUNX2, MMP13, and ALPL activity, but overexpression of OCRL1 blocked these hypertrophic reactions, and reduced mineralization. Besides the mineralization modulators AKT1 and OCRL1, calcium deposition is also regulated by protein kinase C epsilon. In OA chondrocyte micromasses PKCε expression was reduced, resulting in an increase of calcium deposition and calcium crystals and thus increase in the matrix mineralization (Queirolo et al., 2016).

Mineralization is also modulated by MEPE, a matrix mineralization inhibitor (Staines et al., 2016). MEPE was detectable at the lateral OA-unaffected side of STR/Ort mice, while its expression was decreased in the medial affected OA-side of the knee joints. Interestingly, this endochondral phenotype in STR/Ort mice cartilage exists before OA-like changes appear, which suggest that these endochondral changes initiate later development of OA (Staines et al., 2016).

Articular cartilage of many OA patients hosts calcium-containing crystals, which are present in the superficial and deep layers of the cartilage, as well as in synovial fluid and the meniscus (Fuerst et al., 2009; Nguyen et al., 2013). The calcium deposits mainly consist of basic calcium phosphate (BCP), including octacalcium phosphate (OCP), carbonated-apatite (CA), and hydroxyapatite (HA) crystals or calcium pyrophosphate dihydrate (CPPD). Treatment of murine chondrocytes with BCP results in an increase of IL-6 secretion, which in turn induces the expression of pro-mineralizing genes, such as *Ank, Anx5, Pc-1* [plasma-cell membrane glycoprotein 1/*Enpp1* (ectonucleotide pyrophosphatase/ phosphodiesterase 1)] and *Pit1* (Nasi et al., 2016). PC-1, ANK, and TNAPs control extracellular Pi and PPi levels, which are critical determinants of mineralization. Expression of these regulatory proteins is increased in chondrocytes with calcium-containing crystals, forming a positive feedback loop (Nguyen et al., 2013; Nasi et al., 2016).

Another factor involved in OA pathophysiology is Gla-rich protein (GRP). GRP was revealed to inhibit calcification and exerted anti-inflammatory effects in both chondrocytes and synoviocytes (Cavaco et al., 2016). Its expression was increased in OA chondrocytes and synoviocytes compared to non-OA controls. Furthermore, expression of *GRP* was also increased overtime when chondrocyte or synoviocyte mineralization was induced *via* addition of CaCl_2_, evidencing its association with mineralization. Interestingly, GRP was only able to inhibit calcification in its carboxylated form, and since OA chondrocytes and synoviocytes displayed reduced *y*-carboxylation capacity, it was hypothesized that the GRP increase in OA-derived cells is not able to inhibit calcification processes (Cavaco et al., 2016). Besides GRP, MGP, another mineralization inhibitor, has been described as becoming less carboxylated in OA chondrocytes. Lower levels of cMGP lead to a reduction of the fetuin-MGP complex in OA chondrocytes and in released vesicles. This reduction of fetuin-MGP containing vesicles, results in reduced extracellular transport of cMGP, lowering its extracellular concentration. Ultimately, the reduced extracellular cMGP levels lead to an impaired inhibition of matrix mineralization (Wallin et al., 2010).

Taken together, mineralization appears to be an important hallmark of osteoarthritic cartilage and is associated with OA chondrocyte hypertrophy.

## Discussion

The analogs events which are observed during development of OA and endochondral ossification have been described in previous reviews (Dreier, 2010; Pitsillides and Beier, 2011; van der Kraan and van den Berg, 2012). On the contrary, Brew et al. found no evidence of a generalized chondrocyte hypertrophic change in OA (Brew et al., 2010). Despite the lack of evidence found by Brew *et al*., all other papers found in our literature search do describe the endochondral cellular phenotypic changes occurring in OA cartilage. Various signaling pathways and processes involved in endochondral processes, such as Wnt-, Ihh/PTHrP-, TGF-β-, MAP-kinases, FGF-, Notch signaling, inflammatory signaling, and hypoxia-associated signaling pathways, but also processes, such as angiogenesis and matrix mineralization have been described in OA disease progression. Interestingly, from some studies it became evident that crosstalk exists between the different signaling pathways involved in OA disease progression (Ray and Ray, 2008; Papathanasiou et al., 2012; Leijten et al., 2013; van den Bosch et al., 2014; Lin et al., 2016; Zhou et al., 2016). Next to cartilage degradation, which is the main hallmark of OA, it is well established that OA includes changes in several joint structures. Hence, OA should be considered as a total joint disease. Alterations in subchondral bone, meniscal degradation and inflammatory changes in intra-articular tissues, such as the synovium and Hoffa’s fat pad have been widely described. Unraveling mechanisms of feedback and crosstalk among these different joint tissues and their influence on cartilage hypertrophy will further provide important insights in OA pathophysiology.

In this review, we aimed to provide a recent overview of new insights into the contribution of the changing hypertrophic chondrocyte phenotype in the development and progression of OA. In order to include all papers from the past 10 years discussing the endochondral cellular phenotypic changes occurring during OA, we specifically used a search in PubMed which included the term “endochondral ossification,” but also includes terms such as “hypertrophic differentiation,” “hypertrophy,” “mineralization,” and “calcification,” which are all processes occurring during endochondral ossification. Interestingly, different studies use different molecular markers to evaluate the hypertrophic phenotype in the chondrocyte and an unambiguous definition of “hypertrophy” seems lacking (Table 1). This is most likely related to the complexity of the endochondral ossification process with various molecular pathways being involved. In future research, it will be important to reach a consensus and implement a standardized definition to describe “chondrocyte hypertrophy in OA.” We expect that this will support better data comparison between different studies.

A striking realization from our literature search is that it seems that in OA animal models the hypertrophic changes in OA cartilage pathology can be initiated or prevented by a single alteration in many different signaling pathways. While late OA disease progression follows a predictable cell biological progression, little is known about causal relations between the herein described pathways/processes and early OA disease initiation in man. Together this raises the question if OA can even be considered as a generalized single disease (Luyten et al., 2017). Indeed, it is becoming increasingly apparent that different OA phenotypes exist (van der Esch et al., 2015; Deveza et al., 2017; Dell’Isola and Steultjens, 2018). Different early OA phenotypes/disease initiation may result from alterations in molecular pathways that are specifically connected with clinical risk factors for developing OA, like obesity, aging, metabolic syndrome, joint shape/malalignment, or earlier cartilage trauma. We speculate that a phenotyping of early OA, in which dominant deregulation of a specific molecular pathway is taken into account, may provide additional resolution to the already described OA phenotypes. This will not only be important for diagnostic purposes, but equally important for developing new OA disease-modifying compounds and selecting the right patient populations for clinical studies testing novel OA disease-modifying drugs and -approaches.

It can be concluded that articular cartilage/chondrocyte homeostasis is fragile and disruption of chondrocyte homeostasis can be initiated by multiple factors leading to a cascade of intracellular changes in one or multiple pathways. This ultimately results in a chondrocyte hypertrophic/endochondral phenotype that is observed in OA. Understanding the relevance, mechanism-of-action, and crosstalk of processes and signaling pathways involved in OA initiation and progression is expected to further fuel the development of OA disease-modifying drugs and -approaches.

## Author Contributions

All four authors ER, UT, MC, and TW have contributed to literature searches, reading, selecting, and interpretation of papers, and writing of the manuscript. ER and UT have contributed to layout of the tables and MC has contributed to the layout of the figures. The manuscript was approved by all four co-authors prior to submission. ER and UT have contributed equally as first author and MC and TW have contributed equally as last author.

## Conflict of Interest Statement

MC and TW are listed as inventor on filed patents: WO2017178251 and WO2017178253. TW has shares in Chondropeptix BV. The reviewer AL and handling editor declared their shared affiliation.
